# Combining SPECT and Quantitative EEG Analysis for the Automated Differential Diagnosis of Disorders with Amnestic Symptoms

**DOI:** 10.3389/fnagi.2017.00290

**Published:** 2017-09-07

**Authors:** Yvonne Höller, Arne C. Bathke, Andreas Uhl, Nicolas Strobl, Adelheid Lang, Jürgen Bergmann, Raffaele Nardone, Fabio Rossini, Harald Zauner, Margarita Kirschner, Amirhossein Jahanbekam, Eugen Trinka, Wolfgang Staffen

**Affiliations:** ^1^Department of Neurology, Christian Doppler Medical Centre and Centre for Cognitive Neuroscience, Paracelsus Medical University of Salzburg Salzburg, Austria; ^2^Department of Mathematics, Paris Lodron University of Salzburg Salzburg, Austria; ^3^Multimedia Signal Processing and Security Lab, Department of Computer Sciences, Paris Lodron University of Salzburg Salzburg, Austria; ^4^Department of Psychology, Centre for Cognitive Neuroscience, Paris Lodron University of Salzburg Salzburg, Austria; ^5^Spinal Cord Injury and Tissue Regeneration Center, Paracelsus Medical University of Salzburg Salzburg, Austria; ^6^Department of Neurology, Franz Tappeiner Hospital Merano, Italy; ^7^Cardiovascular and Neurological Rehabilitation Center Großgmain, Austria; ^8^Department of Epileptology, University of Bonn Bonn, Germany

**Keywords:** SPECT, EEG connectivity, dementia, depression with cognitive impairment, mild cognitive impairment, subjective cognitive complaints

## Abstract

Single photon emission computed tomography (SPECT) and Electroencephalography (EEG) have become established tools in routine diagnostics of dementia. We aimed to increase the diagnostic power by combining quantitative markers from SPECT and EEG for differential diagnosis of disorders with amnestic symptoms. We hypothesize that the combination of SPECT with measures of interaction (*connectivity*) in the EEG yields higher diagnostic accuracy than the single modalities. We examined 39 patients with Alzheimer's dementia (AD), 69 patients with depressive cognitive impairment (DCI), 71 patients with amnestic mild cognitive impairment (aMCI), and 41 patients with amnestic subjective cognitive complaints (aSCC). We calculated 14 measures of interaction from a standard clinical EEG-recording and derived graph-theoretic network measures. From regional brain perfusion measured by 99mTc-hexamethyl-propylene-aminoxime (HMPAO)-SPECT in 46 regions, we calculated relative cerebral perfusion in these patients. Patient groups were classified pairwise with a linear support vector machine. Classification was conducted separately for each biomarker, and then again for each EEG- biomarker combined with SPECT. Combination of SPECT with EEG-biomarkers outperformed single use of SPECT or EEG when classifying aSCC vs. AD (90%), aMCI vs. AD (70%), and AD vs. DCI (100%), while a selection of EEG measures performed best when classifying aSCC vs. aMCI (82%) and aMCI vs. DCI (90%). Only the contrast between aSCC and DCI did not result in above-chance classification accuracy (60%). In general, accuracies were higher when measures of interaction (i.e., connectivity measures) were applied directly than when graph-theoretical measures were derived. We suggest that quantitative analysis of EEG and machine-learning techniques can support differentiating AD, aMCI, aSCC, and DCC, especially when being combined with imaging methods such as SPECT. Quantitative analysis of EEG connectivity could become an integral part for early differential diagnosis of cognitive impairment.

## 1. Introduction

Mild cognitive impairment (MCI) is common in the elderly population and can be stable or convert to Alzheimer's disease (AD) (Winblad et al., 2004; Gauthier et al., 2006). Estimated 47.5 million people suffer from dementia worldwide, and it is estimated that this number will triple by 2050 (Wold Health Organization, 2016). The WHO reports an estimate of US $604 billion of total global costs associated with dementia. Early differential diagnosis of MCI, subjective cognitive complaints (SCC), and depressive cognitive impairment (DCI) would pave the way for new therapeutic programs, possibly reducing the overall burden of memory disorders and improving quality of life of these patients (DeKosky and Marek, 2003). Because of the various aetiologies and pathologic processes that may lead to memory impairments it is suggested that a combination of several biomarkers is necessary to provide an early diagnosis of AD in the various phases and variations of the disease (Scheltens et al., 1997; DeKosky and Marek, 2003; Wurtman, 2015).

The National Institute of Neurological and Communicative Diseases and Stroke/Alzheimer's Disease and Related Disorders Association (NINCDS-ADRDA) has proposed clinical criteria for the diagnosis of probable AD (McKhann et al., 1984). For an early detection it is not enough to use neuropsychological tests alone since SCC are—by definition—not detectable by these diagnostic procedures, i.e., they are experienced subjectively, only. A patient may suffer from impairment and notice the change. However, a neuropsychological test indicates only whether the patient scores lower than the reference group that was used to standardize the test. When a patient performs well above average throughout his life and experiences a loss because of beginning MCI, he might still perform within the normal range, despite having subjectively noticed the objective decline. In turn, the diagnosis of MCI is still a challenge for neuropsychologists (Ladeira et al., 2009; Lopez, 2013; Rentz et al., 2013). In addition, some of the physiological features that differentiate several types of dementia cannot be assessed with behavioral tests. In the following, we want to outline two diagnostic modalities that might complement each other and thus, are hypothesized to contribute to the differential diagnosis of disorders with amnestic symptoms.

Single Photon Emission Computer Tomography (SPECT) is complementary to clinical assessment (Farid et al., 2011). The measured activity, i.e., the perfusion, can be quantified by volumetric analysis of activated brain regions either manually, semi-automatically, or fully automatically, such as with statistical parametric mapping (SPM) (Friston, 1995; Van Heertum et al., 2009), specifically for differentiating AD from different types of dementia (Kemp et al., 2005). By providing functional information, early stages of cognitive impairment can be identified and differentiation between MCI, AD, and/or other types of cognitive dysfunction can be achieved (Bonte et al., 1990; Talbot et al., 1998; Staffen et al., 2006, 2009; Van Heertum et al., 2009; Farid et al., 2011). Specifically, 99mTc-hexamethyl-propylene- aminoxime (HMPAO)-SPECT seems to be sensitive to cognitive impairment, AD and prodromal stages of AD (e.g., Goldenberg et al., 1989; Frisoni et al., 2014; Swan et al., 2015; Valotassiou et al., 2015). Even when contrasting patients with subjective memory complaints to patients with memory impairment, HMPAO SPECT can be sensitive to cerebral hypoperfusion (Banzo et al., 2011). However, not all studies fully support the usefulness of SPECT for differential diagnosis of disorders with amnestic symptoms (Barnes et al., 2000; Kaneko et al., 2004). Therefore, we suggest combination of SPECT with another physiological modality.

Characteristics from the electroencephalogram (EEG) distinguish patients with AD from MCI and patients with MCI from healthy subjects (see Rossini et al., 2007; Dauwels et al., 2010, for a review). The classical clinical finding is the slow alpha rhythm, which can be quantified as an increase of slow activity; Fast Fourier transform shows a relative increase of activity below 8 Hz and a decrease above this range. The use of the EEG in the assessment of AD dates back to 1952 (see Brenner, 1999, for review). Today it is assumed that the shift toward lower frequencies is possibly caused by perturbations in synchronization and decreased neural complexity (Cantero et al., 2009). Synchronization may be increased or decreased in MCI depending on frequency range, type of analysis, and regions being assessed (Jelic et al., 2000; Koenig et al., 2005; Stam, 2005; Babiloni et al., 2006). Interactions between neural signals are at the forefront of current neuroscientific research, which is also emphasized by the most recent name for this phenomenon: connectomics (Sporns, 2015). The assessment of the connectome has attracted particularly great interest with regard to brain disorders (Fornito et al., 2015). In MCI, interaction between EEG-signals (today, mostly known as connectivity, Aertsen and Preissl, 1991) was found to be a reliable marker for cerebral reserve capacity (Teipel et al., 2016), response to interventions (Klados et al., 2016), and to monitor disease progression (see for recent examples Dimitriadis et al., 2015; Hatz et al., 2015; Wurtman, 2015; Babiloni et al., 2016; Miraglia et al., 2016; Vecchio et al., 2016). Among the plethora of measures indicating interactions between brain regions it is neither clear which ones are preferable over others for diagnostic purposes, nor do we know whether the integration of these measures in to graph-theoretic network characteristics could be a viable method for feature reduction. Therefore, it is recommendable to compare different approaches for characterization of EEG interactions (Lehnertz, 2011). However, because of the low spatial resolution of the EEG, we suggest that it should be combined with neuroimaging in order to yield a full picture of altered brain activity in amnestic disorders.

While it was suggested that the combination of different modalities would contribute to the diagnostic process (Scheltens et al., 1997; DeKosky and Marek, 2003; Wurtman, 2015), only little research was done on the combination of SPECT with EEG. Some studies tried to associate EEG and cerebral perfusion values in patients with AD (Gueguen et al., 1991; Frölich et al., 1992; Sloan et al., 1995). EEG slowing is associated with reduced blood flow in temporo-parietal regions of AD patients (Kwa et al., 1993; Sloan et al., 1994). Degrees of interhemispheric asymmetry of EEG and SPECT are concordant in patients with AD (Montplaisir et al., 1996). Global decrease in cerebral blood flow correlates with a posterior shift of the topographical alpha-centroids (Müller et al., 1997). Power in the EEG delta and alpha frequency ranges correlates with perfusion level in parietal regions and power in the EEG delta range with hippocampal perfusion level of AD patients (Rodriguez et al., 1999). In addition to these correlative studies, some evidence points to a possible complementary use of SPECT and EEG. There is an interaction between alterations in event related potentials recorded with EEG and changes of cerebral blood flow characterized by HMPAO SPECT in AD (Gungor et al., 2005). Specifically, EEG changes take place at earlier stages of the condition than the changes in cerebral blood flow.

No study so far examined the additional value of merging information from advanced EEG measures of interaction and cerebral blood flow measured by SPECT in order to differentiate patients with different types of amnestic syndromes at different stages of AD. We hypothesize that the combined analysis of cerebral perfusion as indicated by HMPAO SPECT and quantitative measures of interaction from the EEG by applying modern methods of data analysis will increase the diagnostic accuracy.

In this study, we assessed the significance of combining EEG measures of interaction or graph-theoretical network characteristics with SPECT perfusion values for differential diagnosis of amnestic SCC (aSCC), amnestic MCI (aMCI), AD, or DCI. Specific expectations about characteristics from the EEG or SPECT that could be most distinctive are restricted to the slowing of the EEG networks, which is more prominent at more advanced stages of cognitive decline, as well as parietal and hippocampal hypoperfusion. Therefore, we applied a machine-learning approach that should identify the most distinctive combination of features from both modalities to pairwise group classifications.

## 2. Materials and methods

### 2.1. Ethics

The study was conducted as a retrospective data analysis. Several years after the examination of the patients had been performed, we analyzed routinely recorded EEG, SPECT, and clinical data. The local Ethics Committee (Ethics Commission Salzburg/Ethikkommission Land Salzburg) confirmed that there are no ethical concerns with respect to this study.

### 2.2. Subjects

We selected 220 consecutive patients from the data repositories at the Department of Neurology, Paracelsus Medical University Salzburg, Austria, which were examined in the memory clinic between June 2007 and March 2011. Diagnosis of aSCC, aMCI, AD, or DCI was assigned at the time of examinations, based on multimodal assessment in the memory clinic of our hospital, including a neurological and neuropsychological examination [German version of the hospital anxiety and depression scale; HADS-D (Zigmond and Snaith, 1983; Herrmann-Lingen et al., 2007), test battery of the Consortium to Establish a Registry for Alzheimer's Disease; CERAD (Morris et al., 1989; Welsh et al., 1994; Thalmann et al., 2000), including a slightly modified version of the mini-mental state examination MMSE by Folstein (Folstein et al., 1975), and in addition (known as the CERAD-Plus tests), the trail making test (Reitan, 1979), and the test for phonematic verbal fluency (Spreen and Benton, 1977)]. The examination included routine laboratory investigations, supplemented by determination of thyroid parameters, internal diagnostics (including electrocardiogram, ECG), cranial computed tomography (CCT), ultrasonographic examination of the carotid and vertebrobasilar arteries, and a cerebral perfusion SPECT scan. The latter was exclusively employed in the differential diagnosis of AD vs. Lewy body dementia, frontotemporal dementia, and vascular dementia based on visually evaluated different patterns of perfusion disturbance. An EEG was recorded in order to disclose epileptic activity.

The diagnosis was assigned by the medical doctor according to the results of the described multimodal examination according to the criteria of Petersen (Petersen et al., 1999). Specifically, we conformed to the definition of aMCI and aSCC where amnestic aMCI equals to level three and patients with aSCC equals to level two of the global deterioration scale for aging and dementia (Winblad et al., 2004; Gauthier et al., 2006). Most importantly, the diagnosis of aMCI and aSCC indicates that the complaints and/or deficits were detectable only in the memory domain, and not on other cognitive subscales.

Patients with DCI were treated with antidepressants after the examinations clarified the diagnosis. However, not all of them were drug-naive at the time of examination since antidepressants are commonly prescribed in the elderly by the general practitioner in order to treat self-reported mood complaints and sleep disorders.

Please note that the diagnosis did not include quantitative assessment of SPECT and EEG as done for the present work. Thus, the original diagnosis of memory impairment was not based on the quantitative analysis as described in the subsequent sections.

### 2.3. SPECT examination

The SPECT examination was performed under quiet conditions (relaxed lying in quiet surroundings and dimmed light 10 min before the injection and during the whole time of the examination), with 99mTc-hexamethyl-propylene- aminoxime (HMPAO, Ceretec, Amersham, UK) serving as perfusion tracer at a dose of 740 MBq. Perfusion was measured 20 min after injection with a three-headed gamma camera (Prism 3000, Picker International, Imaging Division, Cleveland, OH) over 35133815min (3° for 40 steps, i.e., in sum 120°). Datasets were corrected for scatter and attenuation, reconstructed using filtered back projection and displayed as a set of 20 slices using a 128 × 128 matrix. Attenuation correction was applied at the time of reconstruction using Chang's first-order approximation of linear attenuation (μ = 0.09/cm), within an elliptical contour fitted to every slice of the brain (Chang, 1978).

### 2.4. SPECT analysis

For analysis of SPECT data a region of interest (ROI) regionalization was performed automatically to assess relative blood flow (cerebellar ratios) of 46 brain regions. Data were quantified semiautomatically, using the HERMES BRASS Software package (Hermes Medical Solutions, Stockholm, Sweden) which spatially co-registered the image data to an anatomically standardized, stereotactic template consisting of scans of 35 healthy volunteers. Data were count-normalized by the cerebellar count rate and compared to the normal population voxel-by-voxel, as well as on a regional basis. The region map used therefore was predefined using a normal T1-weighted MRI scan co-registered to the normal template.

The regions for which we obtained relative blood flow were cerebellar cortex, cerebellar white matter, nucleus lentiformis, nucleus caudatus, thalamus, sensorimotor cortex, occipital cortex, superior parietal lobule, anterior dorsal frontal region, posterior dorsal frontal region, anterior orbital frontal region, posterior orbital cortex, parietotemporal cortex, medial temporal lobe, lateral temporal lobe, posterior temporal lobe, temporal pole, insular cortex, anterior cingulate gyrus, posterior cingulate gyrus, anterior subcortical region, posterior subcortical region, each of these separately for left and right hemisphere, and in addition one region including pons and midbrain and one region including other subcortical regions. Thus, in sum, the SPECT-feature vector had a length of 46 values.

### 2.5. EEG data registration

EEG was recorded in a quiet room with a clinical standard electrode montage (10–20 Stellate Harmonie Routine EEG System by Natus, 21 channels placed in standard 10–20 EEG system) ground on Fpz, reference on Fcz, with additional earlobe-electrodes for re-referencing, and a sampling rate of 200 Hz. Impedances were kept below 10 kΩ. The EEG recording started with artifact provocation/calibration procedures. Subsequently standard intermittent light stimulation and hyperventilation were performed. Afterwards, the patients were asked to relax with eyes closed.

### 2.6. EEG data extraction

From a period of wakefulness with eyes closed a trained neuroscientist (co-author AL) extracted 3 min of EEG that were free of artifacts, e.g., muscle, eye, movement, etc. Data analysis was conducted for 17 electrodes: F3, F4, C3, C4, P3, P4, O1, O2, F7, F8, T3, T4, T5, T6, Fz, Cz, and Pz. The pre-selected EEG segments were exported into EDF and imported for further processing to Matlab® (release R2016b, The Mathworks, Massachusetts, USA).

### 2.7. Feature extraction

We estimated a set of measures of interaction between all of the 17 selected electrodes (i.e., channels). The estimation was performed for each of the participants. The measures were calculated with the functions mvfreqz.m and mvar.m from the BioSig toolbox (Schlögl and Brunner, 2008) with model order 100 (i.e., equaling half of the sampling rate allowing to model at least one full oscillation beginning from 2 Hz). To estimate the multivariate autoregressive model we used partial correlation estimation with unbiased covariance estimates (Marple, 1987), which was found to be the most accurate estimation method according to Schlögl (2006). The model was then transformed from the time-domain into the *z*-domain and the *f*-domain, which yielded accordingly two transfer functions. The multivariate parameters in the frequency domain that could be derived from these transfer functions were computed for 1 Hz frequency steps between 2 and 80 Hz. The following measures were extracted: auto- and cross-spectrum (S), direct causality (DC), transfer function (h), transfer function polynomial (Af), real valued coherence (COH), complex coherence (iCOH), partial coherence (pCOH), partial directed coherence (PDC), partial directed coherence factor (PDCF), generalized partial directed coherence (GPDC), directed transfer function (DTF), direct directed transfer function (dDTF), full frequency directed transfer function (ffDTF), and Geweke's Granger causality (GGC). A description of these measures and the references can be retrieved in the Supplementary Material Section.

Before statistically determining and evaluating the network characteristics, we averaged the network characteristics in classical frequency ranges delta (2–4 Hz), theta (5–7 Hz), alpha (8–13 Hz), beta (14–30 Hz), and gamma (31–80 Hz).

Finally, we derived graph theoretical measures for each of the listed measures by use of the Brain Connectivity Toolbox (Rubinov and Sporns, 2010). Thus, we calculated global network parameters from the connection matrices in each frequency range obtained from each of the multivariate parameters: assortativity, efficiency, clustering coefficient, modularity, and transitivity. For more details and references of these values we refer to the Supplementary Material.

### 2.8. Feature vectors

Classification and cross-validation was repeated for the following scenarios, which can be described by their respective feature vectors including the following:

1. **EEG single:** each EEG measure individually, that is, feature vector optimization and classification was repeated for each EEG measure, where in every case the initial feature vector was formed as a concatenation of all measures of interaction for each electrode combination and all frequencies
2. **SPECT:** SPECT perfusion values of the 46 brain regions formed the initial feature vector
3. **EEG single** + **SPECT:** a combination of each EEG measure from (1) with the SPECT perfusion values from (2), separately for each EEG measure
4. **EEG merged:** a combination of all optimized feature vectors of the EEG measures from (1) in one feature vector formed the initial feature vector
5. **EEG merged** + **SPECT:** a combination of all optimized feature vectors of the EEG measures from (1) in one feature vector as in (4), combined with the optimized feature vector of SPECT perfusion values from (2) formed the initial feature vector
6. **EEG graph:** a combination of all EEG measures from (1) converted to graph-theoretic measures
7. **EEG graph** + **SPECT:** a combination of all EEG measures from (1) converted to graph-theoretic measures as in (6) combined with the SPECT perfusion values from (2).

### 2.9. Classification analysis

We performed pair-wise classification of all four groups, resulting in 6 group comparisons.

Supervised learning for classification typically includes a training and a testing step, with disjunctive samples for these two steps. That is, the data is divided into two subsets, one is used only for training, and one only for testing according to a defined strategy of cross-validation. The algorithm learns with the training data according to the properties of the samples and their labels, that is, the diagnosis. The result of this learning step is a model that allows to distinguish the members of the groups. In the second step, the algorithm is given only the data of the testing subset, but without the labels. The task is now to predict the correct labels based on the model that was built in the learning step and the data. In order to assess the quality of the classification, the correctness of the predicted labels can be evaluated.

We decided to use support vector machines for classification, because they deal with non-linear properties of the data even when a linear kernel is used. When data are only non-linearly separable, the data is mapped into a feature space in which the linear separating hyperplane can be used. We performed a classification in the sense of supervised learning with a linear kernel function (dot product) and quadratic programming in order to find the separating hyperplane, resulting in a 2-norm soft-margin support vector machine, by using the MATLAB functions svmtrain and svmclassify from the statistics and machine learning toolbox.

### 2.10. Feature subset selection

**Figure 1:**
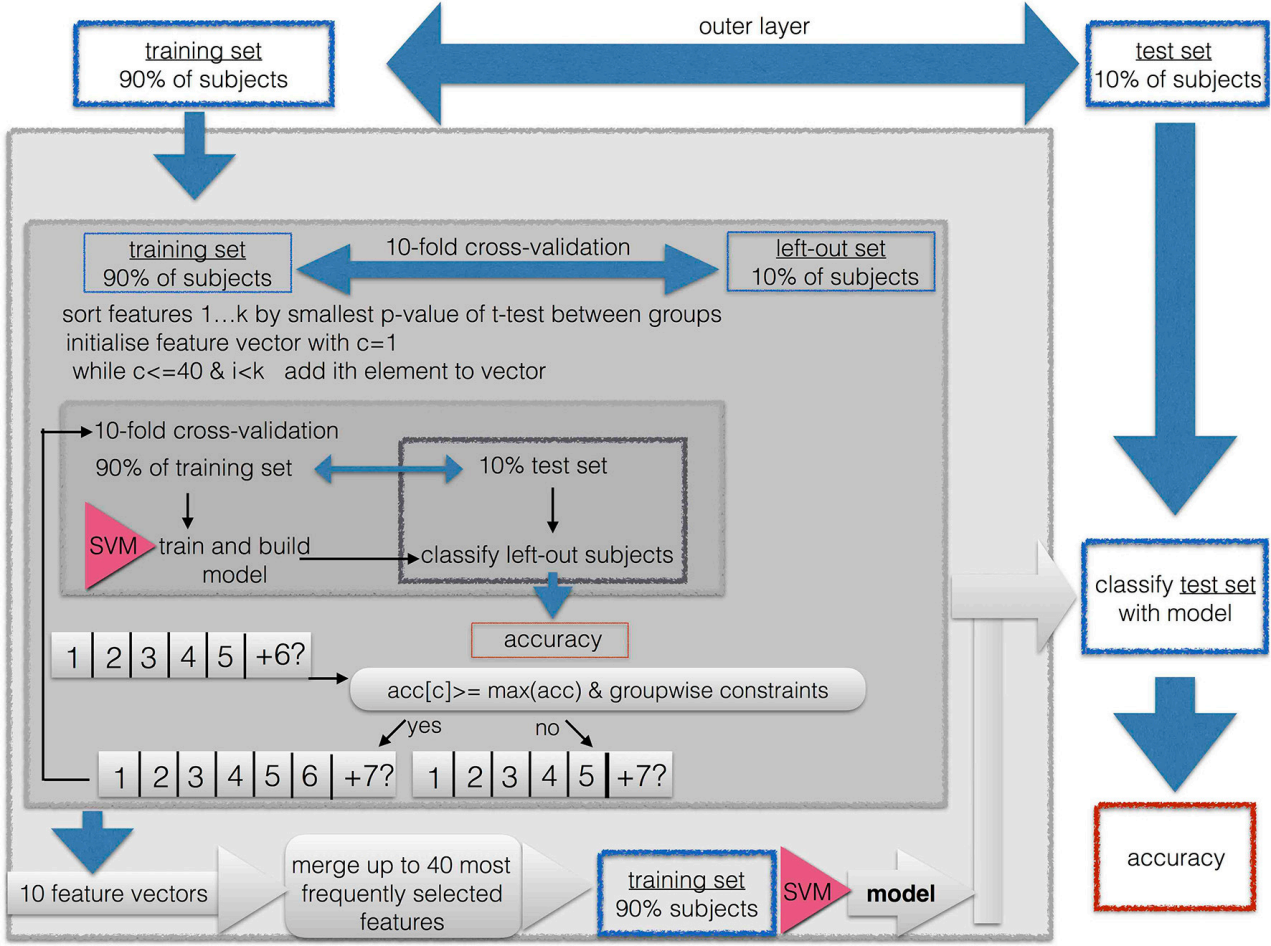
Procedure of cross-validation, classification, and feature subset selection.

We performed a nested cross-validation with 3 layers with feature vector optimization, that is, feature subset selection, for each group comparison as illustrated in Figure 1.

Because of the high dimensionality of the data, we implemented a feature subset selection procedure. This procedure is used for two purposes. First, it is known that when the length of the feature vector exceeds the size of the sample, it can cause artificially high accuracies due to overfitting. Thus, shortening the feature vector to a length that is smaller than the training sample prevents us from running into the small sample size problem. This is easily the case for the EEG feature vectors, because then the length of the feature vector is up to 17 × 17 × 5. Second, a long feature vector with uninformative features prevents the machine learning algorithm from finding a good solution. Therefore, the shortest possible feature vector should be found in the sense of a feature vector optimization. Because the maximally available features for SPECT was 46, we limited the maximally acceptable length of the feature vector to 0.9·46~41 entries. This is well below the smallest sample when combining the two smallest groups of AD (*N* = 39) and aSCC (*N* = 41), where the training sample in the outermost cross validation was 0.9·80 = 72.

As described in Figure 1, the classification and feature subset selection procedure was done in a nested design with 3 layers. We implemented an outer layer as a division of the data into 10% of the data for testing the resulting model, and 90% for feature vector optimization and cross validation, i.e., submitted to the middle layer. The middle layer is a first inner loop, implemented with 10-fold cross-validation. This loop aims to estimate the consistency of selected features, since each run yields a different feature vector. The inner layer is a second, thus, nested inner loop, again with 10-fold cross-validation in order to perform adequate feature subset selection. So-called k-fold cross-validation consist of *k* repetitions of leaving out *N*/*k* samples as the training set, while the remaining *N*−(*N*/*k*) samples are used during the training step.

All subsets were drawn in order to maintain the original proportion of the two groups.

Thus, the whole algorithm can be described as follows:

1. First, 10% of the sample were excluded as the outer-layer test set for the final validation step in the outer layer, while the remaining 90% of patients were used as the outer-layer training set, submitted to the next step
2. The outer-layer training set obtained from the outer loop was divided into 10 equal sized subsets, each one maintaining the proportion of group sizes from the original sample. For each of these 10 sets, the following steps were repeated in order to yield a greedy stepwise feature selection with forward search:
  - This set was left out, the other 9 sets were merged to form the middle-layer training set.
  - A *t*-test for the middle-layer training-set subjects was calculated between the two groups.
  - The resulting *p*-values were sorted in ascending order.
  - The feature vector was initiated by taking the feature with the smallest *p*-value, thus, the initial length was one.
  - For this feature vector, the classification accuracy was calculated with 10-fold cross-validation, thus, the middle-layer training set was divided into an inner-layer 10-fold partition with an inner-layer training- and testing set.
  - The next feature from the sorted list was added. For this feature vector, the inner-layer classification with 10-fold out cross-validation was repeated.
  - The result was compared to the previous result. The added entry to the feature vector was included only if the following three criteria were met:
    - The resulting classification accuracy was required to be at least as high as the maximum of the previously obtained classification accuracies; that is, the second accuracy had to be larger than the first entry, or the 6th accuracy had to be larger than the previous 5 classification accuracies.
    - If the so far best sensitivity/specificity, or in other words, accuracy for members of the first group/second group, respectively, was lower than 0.75, then the obtained sensitivity had to be at least as large as this maximum.
    - If the so far best specificity/sensitivity, was lower than 0.5, then the obtained specificity had to be larger than this maximum.
  - This way, features were added and tested for their contribution to the classification accuracy until all available features were used, or until the feature vector reached a maximum of 40 entries, or if more than a consecutive number of 10% of all available features was not added to the feature vector.
3. The resulting 10 optimized feature sets were concatenated and the occurrences of the features were counted. A final feature vector was formed by including only those features which were selected at least in 3 of the 10 iterations. If this resulted in no features, all features were included that were selected at least in 2 out of 10 iterations, and if this still did not yield any feature, this threshold was lowered to 1 out of 10 iterations. If the resulting feature vector included more than 41 features, only the top-most selected 41 features were included (equaling to approximately 90% of the available SPECT features).
4. The resulting feature vector was used to train a SVM on the outer-layer training set, and the resulting model was used to classify the outer-layer test set, which was then used to calculate the general classification accuracy and the within-group accuracy for the two subgroups (i.e., sensitivity/specificity).

The thresholds of 0.75 and 0.5 were selected as rough estimator for above-chance classification; a value of 0.75 can be considered to be clearly above chance, while values below 0.5 are considered to be clearly below chance and thus, a result of overfitting the model to one of the two groups.

Feature subset selection and classification was done for each of the scenarios as described in Section 2.8 and separately for each of the 6 combinations of groups.

### 2.11. Statistics

We calculated overall group classification accuracy, but also accuracies for the single groups, that can be understood in a sense of sensitivity and specificity. For sensitivity and specificity we have to define what are the positives and what the negatives, which is not directly applicable to pairwise group classifications. Thus, the accuracy of the single groups was

(1) $$\begin{array}{r} acc_{group}=\frac{\text{N correct in group}}{\text{total N of group}} \end{array}$$

Namely, for each group the proportion of correctly classified individuals was determined in each of the classification situations (feature vectors and group combinations).

In order to evaluate the resulting accuracies we calculated the maximum-chance criterion, that is the proportion of samples contained in the larger of the two groups of one group comparison.

Wilcoxon-tests, *t*-tests, or Fisher's exact tests were used as appropriate for pairwise group-comparisons of numerical or nominal data characteristics of the samples. We applied Bonferroni correction to the resulting *p*-values by interpreting them at the level 0.05/(16^*^6) for the 6 group comparisons and 16 neuropsychological scales and demographic aspects.

## 3. Results

### 3.1. Sample details

The demographic details as well as the results of the neuropsychological scales of the patients are given group-wise in Table 1.

**Table 1:** Sample overview with means and standard deviations in parentheses for neuropsychological test results.

**Sample**	**aSCC**	**aMCI**	**AD**	**DCI**
*N*	41	71	39	69
Median age	68	70	76	69
Mean age	67.54	70.04	74.97	68.81
Age range	52–82	51–87	57–90	50–86
N women	30	38	27	51
**HADS**				
Anxiety (SD)	52.46 (10.30)	50.72 (11.61)	50.91 (11.27)	61.45 (9.21)
Depression (SD)	53.54 (5.66)	53.68 (8.82)	55.15 (8.85)	71.18 (6.60)
**CERAD**				
Semantic verbal fluency *z*-value (SD)	0.30 (0.90)	−0.60 (0.90)	−1.73 (0.86)	−0.50 (1.15)
Boston naming test *z*-value (SD)	0.49 (0.80)	−0.26 (1.06)	−1.09 (1.50)	−0.21 (1.35)
MMSE raw (SD)	28.66 (1.06)	26.80 (1.66)	20.23 (4.50)	27.81 (1.62)
Wordlist learning *z*-value (SD)	−0.51 (0.97)	−1.67 (0.89)	−3.31 (1.28)	−1.03 (1.24)
Wordlist recall *z*-value (SD)	−0.37 (0.64)	−1.60 (0.91)	−2.78 (0.93)	−0.77 (0.98)
Wordlist recognition *z*-value (SD)	−0.10 (0.91)	−1.46 (1.30)	−2.56 (1.41)	−0.40 (1.41)
Figures copying *z*-value (SD)	0.50 (0.94)	0.13 (1.14)	−1.29 (1.95)	0.50 (1.18)
Figures recall *z*-value (SD)	0.55 (1.19)	−1.08 (1.33)	−2.16 (1.02)	0.32 (1.53)
Figures recognition *z*-value (SD)	0.36 (1.03)	−0.90 (1.21)	−1.69 (1.23)	0.03 (1.15)
**PLUS-TESTS**				
Trail making test A *z*-value (SD)	0.81 (1.06)	−0.16 (1.18)	−1.73 (1.28)	−0.04 (1.54)
Trail making test B *z*-value (SD)	0.48 (1.27)	−0.32 (1.11)	−1.25 (0.83)	0.31 (1.21)
Phonematic verbal fluency *z*-value (SD)	0.44 (0.99)	0.08 (1.07)	−0.55 (1.15)	−0.05 (1.25)

*N, number; aSCC, amnestic subjective cognitive complaints; aMCI, mild cognitive impairment*.

*AD, Alzheimer's disease; DCI, depression with cognitive impairment; SD, standard deviation*.

*z-values of the CERAD scores refer to the relative scores with respect to a normative (cognitively healthy). group and adjusted for age and education*.

The results of the pairwise group comparisons are shown in Table 2.

**Table 2:** Sample comparisons test-value/*p*-values.

**Comparison**	**aSCC-aMCI**	**aSCC-AD**	**aSCC-DCI**	**aMCI-AD**	**aMCI-DCI**	**AD-DCI**
Age (*t*-test)	−1.55/0.12	−4.15/^*^	−0.81/0.42	−2.79/0.006	0.84/0.40	3.56/<0.001
Sex (Fisher)	2.37/0.05	1.21/0.81	0.96/1	0.51/0.16	0.41/0.01	0.79/0.66
**HADS**						
Anxiety (Wilc)	0.78/0.43	0.94/0.35	−4.52/^*^	0.14/0.89	−5.48/^*^	−4.76/^*^
Depression (Wilc)	0.26/0.80	−1.02/0.31	−8.39/^*^	−1.11/0.27	−8.80/^*^	−7.19/^*^
**CERAD**						
Semantic vf (Wilc)	4.35/^*^	6.98/^*^	3.54/^*^	5.36/^*^	−0.66/0.51	−5.33/^*^
Boston naming test (Wilc)	3.66/^*^	4.90/^*^	2.48/0.01	2.92/0.004	−0.50/0.62	−2.97/0.003
MMSE (Wilc)	5.55/^*^	7.74/^*^	2.54/.01	8.17/^*^	−3.71/^*^	−8.37/^*^
Wordlist learning (Wilc)	5.42/^*^	7.15/^*^	1.99/.05	6.07/^*^	−3.65/^*^	−6.87/^*^
Wordlist recall (Wilc)	6.76/^*^	7.36/^*^	1.87/.06	5.48/^*^	−5.05/^*^	−7.31/^*^
Wordlist recognition (Wilc)	5.52/^*^	6.22/^*^	1.14/.26	3.91/^*^	−4.50/^*^	−5.89/^*^
Figures copying (Wilc)	1.73/0.08	4.07/^*^	−0.90/0.37	3.74/^*^	−2.49/0.01	−4.40/^*^
Figures recall (Wilc)	5.48/^*^	7.11/^*^	0.72/0.47	4.35/^*^	−4.94/^*^	−6.80/^*^
Figures recognition (Wilc)	5.04/^*^	6.15/^*^	1.28/.20	3.52/^*^	−4.27/^*^	−6.00/^*^
**PLUS-TESTS**						
Trail making test A (Wilc)	3.92/^*^	6.24/^*^	2.87/.004	5.03/^*^	−0.55/.58	−4.87/^*^
Trail making test B (Wilc)	2.91/0.004	3.98/^*^	0.39/0.70	2.66/0.008	−2.74/0.006	−3.65//^*^
Phonematic vf (Wilc)	1.63/0.10	3.29/<0.001	1.78/0.07	2.34/0.02	0.21/0.83	−2.08/0.04

vf, verbal fluency; Fisher's test, oddsRatio/p-value; t-test, t-value/p-value; Wilc: Wilcoxon, z-value/p-value;

aSCC, amnestic subjective cognitive complaints; aMCI, mild cognitive impairment; AD, Alzheimer's disease;

DCI, depression with cognitive impairment;

* *significant at Bonferroni-corrected level p < 0.00052083*.

### 3.2. Classification results

The results of the classification are given in Table 3. We marked the best classification accuracies in bold font, where the best accuracy was defined as the highest overall accuracy and also high within group accuracies. We can see that for all comparisons involving AD, that is, aSCC-AD, aMCI-AD, and AD-DCI, the best result was obtained when combining a single EEG measure with SPECT. For the comparisons of aSCC-aMCI and aMCI-DCI the best comparison was obtained when merging all EEG measures, and adding SPECT to this configuration yielded the same result. For the comparison aSCC-DCI the best result was found for single EEG measures, but the accuracies were below the maximum-chance criterion.

**Table 3:** Sample comparison classification accuracies.

	**aSCC-aMCI**	**aSCC-AD**	**aSCC-DCI**	**aMCI-AD**	**aMCI-DCI**	**AD-DCI**
**EEG SINGLE**						
DC	0.36(0.5/0.29)	0.6(0.6/0.6)	**0.6(0.67/0.57)**	0.5(0.5/0.5)	0.5(0.4/0.6)	0.6(0/1)
S	0.64(0.75/0.57)	0.8(0.8/0.8)	0.2(0.67/0)	0.5(0.83/0)	0.5(0.4/0.6)	0.8(0.5/1)
h	0.73(0.5/0.86)	0.5(0.6/0.4)	**0.6(0.67/0.57)**	0.6(0.5/0.75)	0.8(0.8/0.8)	0.7(0.75/0.67)
Af	0.55(0.25/0.71)	0.3(0/0.6)	0.2(0/0.29)	0.6(0.5/0.75)	0.7(0.8/0.6)	0.5(0.75/0.33)
COH	0.55(0.75/0.43)	0.8(0.8/0.8)	0.2(0.67/0)	0.6(0.33/1)	0.6(0.2/1)	0.7(0.25/1)
iCOH	0.36(0.5/0.29)	0.5(0.6/0.4)	0.5(0.33/0.57)	0.5(0.5/0.5)	0.7(0.4/1)	0.5(0.25/0.67)
pCOH	0.55(0.5/0.57)	0.7(0.8/0.6)	0.4(0.67/0.29)	0.5(0.5/0.5)	0.5(0.6/0.4)	0.7(0.5/0.83)
PDC	0.64(0.75/0.57)	0.7(0.6/0.8)	0.5(0.67/0.43)	0.5(0.83/0)	0.5(0.4/0.6)	0.6(0.25/0.83)
PDCF	0.27(0.25/0.29)	0.8(1/0.6)	0.6(0.33/0.71)	0.4(0.33/0.5)	0.6(0.4/0.8)	0.6(0.75/0.5)
GPDC	0.36(0.75/0.14)	0.5(0.8/0.2)	0.6(0.33/0.71)	0.3(0.5/0)	0.5(0.4/0.6)	0.6(0.5/0.67)
DTF	0.18(0.25/0.14)	0.5(0.8/0.2)	0.3(0.67/0.14)	0.5(0.67/0.25)	0.8(0.8/0.8)	0.5(0.5/0.5)
dDTF	0.27(0.25/0.29)	0.5(0.4/0.6)	0.5(0.67/0.43)	0.5(0.83/0)	0.8(1/0.6)	0.5(0.25/0.67)
ffDTF	0.55(0.75/0.43)	0.4(0.4/0.4)	0.4(0.67/0.29)	0.4(0.5/0.25)	0.6(0.2/1)	0.6(0.25/0.83)
GGC	0.36(0.5/0.29)	0.6(0.4/0.8)	0.6(0.33/0.71)	0.4(0.33/0.5)	0.5(0.2/0.8)	0.6(0.25/0.83)
SPECT	0.18(0/0.29)	0.8(1/0.6)	0.4(0.33/0.43)	0.5(0.33/0.75)	0.5(0.6/0.4)	0.7(0.75/0.67)
**EEG SINGLE** + **SPECT**						
DC + SPECT	0.45(0.25/0.57)	**0.9(1/0.8)**	0.5(0.33/0.57)	0.6(0.5/0.75)	0.5(0.4/0.6)	0.7(0.75/0.67)
S + SPECT	0.64(0.75/0.57)	**0.9(1/0.8)**	0.3(0/0.43)	0.5(0.33/0.75)	0.5(0.4/0.6)	0.8(0.75/0.83)
h + SPECT	0.73(0.5/0.86)	0.8(1/0.6)	0.6(0/0.86)	**0.7(0.83/0.5)**	0.8(0.8/0.8)	**1(1/1)**
Af + SPECT	0.55(0.25/0.71)	0.8(0.8/0.8)	4(0/0.57)	**0.7(0.67/0.75)**	0.7(0.8/0.6)	0.7(0.75/0.67)
COH + SPECT	0.55(0.75/0.43)	**0.9(1/0.8)**	0.6(0.33/0.71)	0.6(0.5/0.75)	0.6(0.2/1)	0.6(0.5/0.67)
iCOH + SPECT	0.55(0.5/0.57)	**0.9(1/0.8)**	0.5(0/0.71)	0.5(0.5/0.5)	0.8(0.6/1)	0.7(0.5/0.83)
pCOH + SPECT	0.55(0.5/0.57)	0.7(0.8/0.6)	0.5(0.67/0.43)	0.6(0.5/0.75)	0.5(0.6/0.4)	0.9(0.75/1)
PDC + SPECT	0.64(0.75/0.57)	0.7(1/0.4)	0.4(0/0.57)	0.5(0.33/0.75)	0.5(0.4/0.6)	0.7(0.5/0.83)
PDCF + SPECT	0.27(0.25/0.29)	**0.9(1/0.8)**	0.4(0/0.57)	0.5(0.5/0.5)	0.6(0.4/0.8)	0.7(0.75/0.67)
GPDC + SPECT	0.36(0.75/0.14)	**0.9(1/0.8)**	0.4(0/0.57)	0.6(0.5/0.75)	0.5(0.4/0.6)	0.7(0.75/0.67)
DTF + SPECT	0.18(0.25/0.14)	0.7(0.8/0.6)	0.4(0.67/0.29)	0.5(0.5/0.5)	0.8(0.8/0.8)	0.8(0.75/0.83)
dDTF + SPECT	0.27(0.25/0.29)	0.6(0.4/0.8)	0.5(0.67/0.43)	0.4(0.33/0.5)	0.8(1/0.6)	0.8(0.5/1)
ffDTF + SPECT	0.55(0.75/0.43)	0.7(0.8/0.6)	0.5(0.33/0.57)	0.5(0.33/0.75)	0.6(0.2/1)	0.6(0.25/0.83)
GGC + SPECT	0.36(0.5/0.29)	**0.9(0.8/1)**	0.6(0.33/0.71)	0.4(0.33/0.5)	0.5(0.2/0.8)	0.7(0.25/1)
EEG merged	**0.82(0.75/0.86)**	0.8(0.6/1)	0.6(0.33/0.71)	0.4(0.67/0)	**0.9(1/0.8)**	0.6(0.25/0.83)
EEG merged + SPECT	**0.82(0.75/0.86)**	0.7(0.4/1)	0.6(0.33/0.71)	0.5(0.67/0.25)	**0.9(1/0.8)**	0.6(0.25/0.83)
EEG graph	0.55(0.75/0.43)	0.7(0.4/1)	0.2(0.33/0.14)	0.5(0.67/0.25)	0.4(0.2/0.6)	0.7(0.5/0.83)
EEG graph + SPECT	0.55(0.75/0.43)	0.8(0.8/0.8)	0.4(0.67/0.29)	0.6(0.67/0.5)	0.4(0.2/0.6)	0.8(0.75/0.83)
Chance level	0.63	0.65	0.63	0.65	0.51	0.64

*aSCC, amnestic subjective cognitive complaints; aMCI, amnestic mild cognitive impairment; AD, Alzheimer's disease; DCI, depression with cognitive impairment; bold font, best result; S, auto- and cross-spectrum; DC, direct causality; h, transfer function; Af, transfer function polynomial; COH, real valued coherence; iCOH, complex coherence; pCOH, partial coherence; PDC, partial directed coherence; PDCF, partial directed coherence factor; GPDC, generalized partial directed coherence; DTF, directed transfer function; dDTF, direct directed transfer function; ffDTF, full frequency directed transfer function; GGC, Geweke's Granger causality; chance level, maximum-chance criterion according to maximum of group proportions*.

Please note that, however, the combination leads to a re-ordering of the features during the sorting according to *p*-values, so that merging of EEG and SPECT does not necessarily mean that there were actually features from the EEG or SPECT included in the analysis. For example, the classification accuracy for aSCC vs. AD was already quite high when using SPECT alone. When introducing the EEG measure spectrum, none of the EEG measures was finally used, but the additional features in the feature vector helped to choose the most informative SPECT values so that the accuracy was higher.

### 3.3. Visualization of group differences

For clinical interpretability we created heatmaps for all measures. Since EEG+SPECT yielded most informative measures, we based this illustration on the features selected from this combination. The heatmaps represent *t*-values for the pairwise group comparisons of the EEG, where all non-used indices were set to zero. Thus, we highlighted the region-interactions/frequencies that were selected during feature subset selection. In addition, we noted which SPECT regions were included into the analysis.

We include here only two measures as examples, while the others can be retrieved in the Supplementary Material Section. We include transfer function (h) which is the base on which the other measures are calculated, and which indeed yields reasonable accuracies for several comparisons.

**Figure 2:**
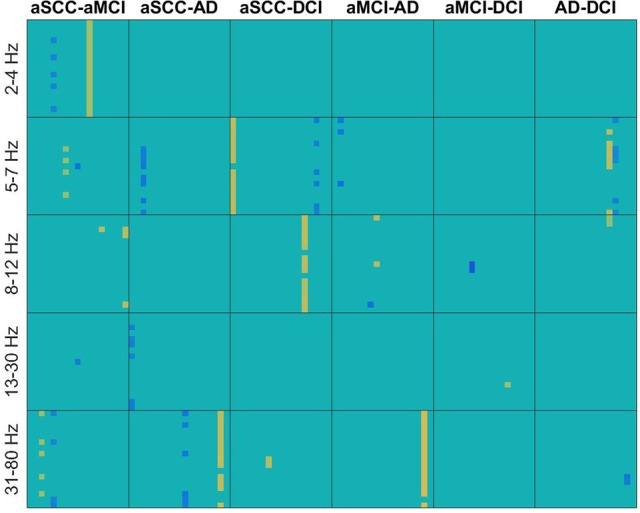
Heatmaps of the *t*-values of group differences of all electrode × electrode interactions for transfer function, sorted by groups comparisons in columns and frequency ranges in rows. Colors indicate values from −4.11 (dark blue) over zero (green) to +5.24 (yellow). All values that were not included for classification were set to zero. If the first group of the group comparison (e.g., aSCC in aSCC-aMCI) has higher values than the second group, this results in a positive *t*-value, i.e., yellow colors. Electrodes start from top to bottom and from left to right following the order: F3, F4, C3, C4, P3, P4, O1, O2, F7, F8, T3, T4, T5, T6, Fz, Cz, and Pz. AD, Alzheimer's disease; DCI, depression with cognitive impairment; aMCI, mild cognitive impairment with amnestic symptoms; aSCC, subjective cognitive complaints with amnestic symptoms.

From Figure 2 we can see that from the transfer function polynomial, single channels are selected because of the information spread from these channels toward others. For most comparisons, the classifier was based on at least one such interaction where the strength was higher in the one than in the other group and at least one such interaction with the opposite pattern.

**Figure 3:**
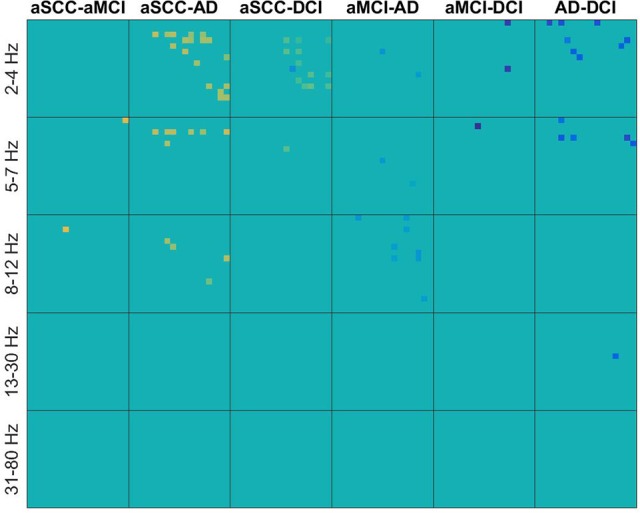
Heatmaps of the *t*-values of group differences of all electrode × electrode interactions for real valued coherence, sorted by groups comparisons in columns and frequency ranges in rows. Colors indicate values from −4.11 (dark blue) over zero (green) to +5.24 (yellow). All values that were not included for classification were set to zero. If the first group of the group comparison (e.g., aSCC in aSCC-aMCI) has higher values than the second group, this results in a positive *t*-value, i.e., yellow colors. Electrodes start from top to bottom and from left to right following the order: F3, F4, C3, C4, P3, P4, O1, O2, F7, F8, T3, T4, T5, T6, Fz, Cz, and Pz. AD, Alzheimer's disease; DCI, depression with cognitive impairment; aMCI, mild cognitive impairment with amnestic symptoms; aSCC, subjective cognitive complaints with amnestic symptoms.

In contrast, in Figure 3 we can see for real valued coherence the typical pattern of information contained in the lower frequencies, where patients with aSCC showed higher values than the other groups, followed by DCI and then AD. Single electrode interactions were chosen, and most information was contained in the frequency ranges delta, theta and alpha, while beta contributed only with a single value for AD vs. DCI and the gamma range was not informative, at all.

**Figure 4:**
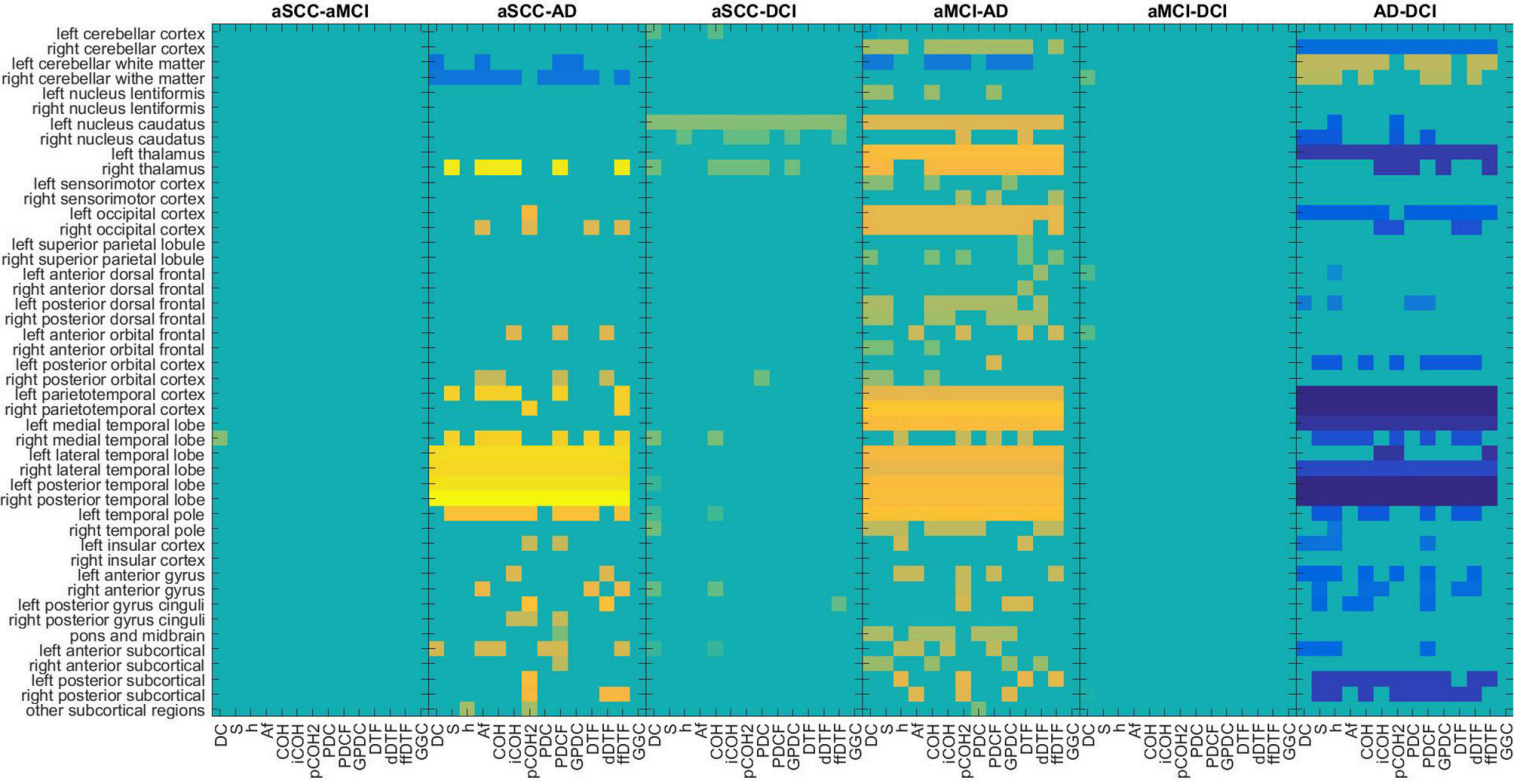
Maps of the *t*-values of group differences of all regions of interest assessed by SPECT perfusion, sorted by groups comparisons, colored according to use for classification in combination with EEG measures. Colors indicate values from −4.95 (dark blue) over zero (green) to +6.07 (yellow). All values that were not included for classification were set to zero. If the first group of the group comparison (e.g., aSCC in aSCC-aMCI) has higher values than the second group, this results in a positive *t*-value, i.e., yellow colors. For each measure (columns of subplots) only those regions (rows of subplots) were colored according to the *t*-values that were used for classification. AD, Alzheimer's disease; DCI, depression with cognitive impairment; aMCI, mild cognitive impairment with amnestic symptoms; aSCC, subjective cognitive complaints with amnestic symptoms.

The regions typically used from SPECT (Figure 4) are quite consistent across the EEG measures, especially for the comparisons with the AD group. Patients with AD have lower perfusion values in bilateral parietotemporal cortex, medial, lateral, and posterior temporal-lobe, and the temporal pole. In addition, differences in the cerebellum (cortex and white matter), the occipital cortex, and the thalamus were useful sources for information. However, while all the regions mentioned here were found to show lower perfusion values in AD than all other groups, the cerebellar white matter evokes higher values in AD compared to DCI.

## 4. Discussion

In this work, we examined the diagnostic accuracy of quantitative EEG and SPECT alone and in combination with each other in order to differentiate patients with AD, aMCI, aSCC, and DCI. SCC are common in the elderly population and can be an early phase of MCI (Kryscio et al., 2014). Patients with SCC are twice as likely to develop AD than people without SCC (Mitchell et al., 2014). Conversion rates of MCI to AD are estimated around 10–18% per year (Gauthier et al., 2006), 11–33% after 2 years (Ritchie, 2004), and 50–70% after 3–5 years (see review in Rossini et al., 2007). Depressive symptoms in the elderly affect daily living and severely reduce quality of life (Stögmann et al., 2016a). Depressive symptoms correlate with conversion from MCI to AD (Makizako et al., 2016; Stögmann et al., 2016b), and can challenge differential diagnosis (Leyhe et al., 2017). Early differential diagnosis between these disorders with amnestic symptoms is a prerequisite to targeted interventions.

We found that for specific comparisons, a combination of EEG and SPECT yields the best diagnostic accuracy, while for other group contrasts, the one or the other modality is superior. In the following, we want to discuss our results in relation to previously reported classification approaches and we want to emphasize the novelty of a possible classification of DCI by using EEG and SPECT in combination.

### 4.1. EEG—an underestimated source of information?

Previous research has suggested that biomarkers from the EEG may be more useful than methods investigating cerebral perfusion, such as HMPAO SPECT in order to identify patients suffering from AD at an early stage of the condition (Gungor et al., 2005). Still, the additional contribution of EEG seems to be underestimated, since EEG alterations such as slow theta-delta activity are a common feature of dementia and natural aging, as well (Rossini et al., 2007). In our study, alterations in the delta, theta, and alpha frequency ranges were prominent when comparing patients with AD to the other groups in widespread regions, where the exact localization of the most informative region depended highly on the measure of interaction. In patients with AD, coherence was lower than in patients with SCC, and than in patients with DCI, while patients with aMCI showed lower coherence than patients with AD. Previous research reported that within and between hemisphere alpha coherence values are reduced in patients with dementia that show abnormal regional cerebral blood flow (Sloan et al., 1994). We could extend this finding by showing directly that combination of measures of interaction, for example partial coherence, with SPECT provides considerable information gain in a differential diagnostic setting. However, our results also demonstrate that the clear findings reported in the literature depend highly on the choice of the measure.

We want to mention that we performed a rather simple feature merging algorithm, and also the feature subset selection technique presented here is not able to fully explore the information in the data. In order to reduce computational complexity, the feature vectors were sorted by *p*-values. Processing the features in a different order might have yielded different results, which is also emphasized by the case when adding SPECT to EEG spectrum changes the results, even when the information from SPECT might not have been used (as found for spectrum). With more sophisticated feature subset selection techniques and feature merging algorithms we might achieve even higher accuracies.

The largest difference between information content in EEG and SPECT is seen for aSCC vs. DCI, where the best result is obtained with EEG-measures, only. However, the resulting accuracies are at chance, so that it is likely that none of the two modalities is able to accurately differentiate these two disorders. In contrast, the comparison of aSCC vs. aMCI and aMCI vs. DCI was highest when the best features from all EEG measures were merged, where this result did not change when including SPECT to the feature vector. The evidence for SPECT being useful to identify SCC or aSCC is scarce (Banzo et al., 2011; Frisoni et al., 2014). The differential diagnosis of aSCC is a challenge. In our study, we included patients with minimal deviations on the neuropsychological scales for memory, but who did not yet fulfill the clinical criteria for aMCI. Nevertheless, whether aSCC is a state of normal aging, where the patients become aware of the natural decay of memory capacities, or whether this is the first sign of a beginning dementia cannot be determined by neuropsychological scales, unless one has longitudinal data at his disposal. The group in our study may be very heterogeneous, for these reasons. On this background it is remarkable that we were able to report above-chance classification accuracies of the EEG biomarkers.

EEG also successfully differentiated DCI from aMCI, best when merging all EEG measures, and from AD, combination with SPECT, yielding reasonable classification accuracies. Only the comparison of aSCC vs. DCI was not above chance with none of the applied feature vectors. A similar classification experiment of DCI vs. AD, aMCI and aSCC was—to our best knowledge—never done before with EEG, so that this result points to a new field of application. Especially in aMCI or AD depression is not rare and the differential diagnosis is often based on the trend of the symptoms when treating the depression adequately. Cognitive improvement after antidepressive therapy suggests that the depression, not a neurodegenerative disorder, causes the symptoms. As a conclusion the diagnosis of DCI can be made. However, since dementia and depressive symptoms coexist in some cases it could be difficult to assess whether depression is the cause or the effect of the cognitive impairment and vice versa. This is especially true when considering that depression is suspected to play a role in the progression of aMCI to AD (Van der Mussele et al., 2014; Chung et al., 2016).

Using robust invariant features from unprocessed EEGs, it may even be possible to reach higher classification accuracies than in the present manuscript (Buscema et al., 2015; Dimitriadis et al., 2015). However, in our study we used strict nested cross-validation, which is the state of the art in order to avoid overfitting during parameter selection, and could rely on our sample with a sufficient size without need for data augmentation techniques as implemented in other studies (Dimitriadis et al., 2015). Moreover, the intention of this study was not to reach maximum classification accuracy of one particular method, but rather to show how EEG and SPECT could complement each other, while trying to render the comparison between individual and combined methods as fair as possible. However, our results are comparable with previous publications (Buscema et al., 2015; Gallego-Jutgla et al., 2015; Hatz et al., 2015). Other studies using entropy measures instead of measures of interaction report results with accuracies of 91.7–93.8% when discriminating MCI, AD and normal controls (McBride et al., 2015). After all, there was no healthy control group in our study, and the comparison to healthy controls is more straightforward and clinically not of interest, because differential diagnosis between AD and healthy or even aSCI can be accomplished reliably with classical paper and pencil tests. In contrast, we examined also the more challenging and interesting discrimination of DCI vs. AD or vs. aMCI yielding excellent classification accuracies.

### 4.2. Information gain or information loss through graph-theory

It was suggested that graph-theoretical approaches could help to make measures of interaction more useful for the prediction of MCI progression from the EEG (Vecchio et al., 2014, 2015; Miraglia et al., 2016; Rossini et al., 2016; Vecchio et al., 2016). In our study, using the measures of interaction directly yielded higher accuracies than the use of the derived graph-theoretic indices. Only for aSCC vs. AD and for AD vs. DCI above-chance classification (0.8) was obtained with graph-theoretical measures. This means that the way the information is integrated with graph-theoretical measures may not be advantageous in every scenario and needs to be examined from case to case.

### 4.3. HMPAO-SPECT

A systematic review found sensitivity and specificity of HMPAO-SPECT to distinguish AD from healthy controls to be 76.1 and 85.4%, respectively, and the distinction of vascular dementia and dementia with Lewy Bodies from AD yielded even lower diagnostic values (Yeo et al., 2013). We want to emphasize that when contrasting HMPAO-SPECT of AD and healthy controls, sensitivities and specificities are high: 81 and 96% (Fleming et al., 2002), or 91 and 86% (Johnson et al., 1993). However, when cases with diagnostic uncertainty are examined, only very low values with a sensitivity of 71–77% and a specificity of 38–44% can be achieved (Doran et al., 2005). It is also hard to identify AD among unselected patients in a memory clinic, resulting in a sensitivity of 75% and a specificity of 52% (Masterman et al., 1997). Indeed, a systematic review found that the diagnostic accuracy of HMPAO-SPECT to discriminate between AD and other forms of dementia was characterized by a sensitivity of 71.3% and a specificity of 75.9% (Dougall et al., 2004a). This is also reflected by our results, where the highest accuracy values when using the SPECT-feature vector, only, were found for aSCC vs. AD. In clinical terms, this is the most obvious differentiation, followed by the more challenging contrasts of AD vs. DCI and then by AD vs. aMCI. There is a statistically significant difference in perfusion in specific brain areas between AD and aMCI (Fröhlich et al., 1989; Staffen et al., 2006, 2009; Tranfaglia et al., 2009; Van Heertum et al., 2009; Farid et al., 2011), but according to our results, it is not enough for creating a model with high distinctiveness when being used without further information, such as the EEG. It is worth to stress once again that our results were based on a quantitative evaluation, while many of the diagnostic characteristics of SPECT are based on expert ratings. The sensitivity of these ratings was found to be negatively correlated with the importance the expert attributed to regional hypoperfusion in the parietal lobes (Dougall et al., 2004b).

The contribution of HMPAO SPECT to the differentiation of DCI and other forms of cognitive impairment is well in line with the finding, that depression and specifically treatment-resistant depression shows significant alterations in circumscribed brain regions such as the hippocampus and the amygdala (Bonne et al., 1996; Mozley et al., 1996; Hornig et al., 1997; Kowatch et al., 1999; Cho et al., 2002). In patients with AD and depression, a selective hypoperfusion in the anterior and posterior cingulate gyrus and in the precuneus was reported (Liao et al., 2003). A direct comparison between patients with AD and DCI showed differences in perfusion in the left parieto-occipital lobe (Stoppe et al., 1995). Thus, it is likely that the contribution of SPECT to EEG can be explained by complementary information about regional abnormalities in DCI that differ from those of AD. Indeed, the regions that differ between aMCI and AD are also most informative when differentiating AD from DCI. Future work should have a closer look at the distinctive characteristics of DCI, where only a narrow range of publications have identified promising biomarkers.

### 4.4. Limitations

Firstly, this retrospective study cannot indicate which markers are important for prognostic questions. Nevertheless, prognosis is the most important question in this patient population. Therefore, future studies with longitudinal, prospective design are needed to clarify the role of EEG and SPECT in these respects.

Secondly, the ground truth of our sample is based on multimodal clinical assessment. That is, we have no post-mortem determination of definitive AD. This implies that the ground truth is somewhat unclear and that the diagnoses that were used for classification are not all correct. In addition, this means that SPECT and sometimes also EEG were part of the basis on which the clinician defined the diagnosis, which is in turn, our ground truth. This is the typical scenario in the clinics, but still, a drawback of retrospective studies. However, as described in Section 2.2, the EEG examination was not used to define one of the examined diagnoses, but to disclose epilepsy or other disorders that could cause the amnestic symptoms. Similarly, SPECT was only included in the diagnostic process for differential diagnosis of disorders that were not included in the presented analysis. Moreover, the examination of EEG and SPECT at the time of diagnosis was performed only qualitatively, while the present work was based on quantitative analysis, only. In sum, we estimate the bias in our ground truth to be very small.

Third, the present study emphasizes that the EEG can be useful at the stage of aSCC. However, our study did not provide data from a healthy control group, mainly because it is difficult to obtain SPECT from healthy controls. Future studies using EEG will more easily recruit healthy controls and provide longitudinal data. The latter is important in order to demonstrate the predictive value of the identified biomarkers.

Fourth, we could not report the medication history of the patients but we assume that only a minority of them were drug-naive at the time of examination. Specifically antidepressants are commonly prescribed in the elderly and it is possible that they are prescribed more likely in the group of DCI, since these patients might have consulted the general practitioner before visiting the memory clinic.

Finally, there are other diagnostic modalities such as structural MRI which show a very high diagnostic accuracy and increasing relevance in amnestic populations (Teipel et al., 2013). However, the purpose of this study was not to show the best method in order to contribute to the diagnosis, but to show whether the combination of EEG and SPECT is a valid approach. Especially EEG is a cheap and one of the most easily available diagnostic methods that could be integrated into the routine process of memory clinics.

## 5. Conclusions and future directions

HMPAO SPECT alone cannot reliably identify AD and related disorders with memory problems, but its additive value in combination with other modalities is well acknowledged. Also the examination of the EEG has identified several useful biomarkers that could be considered for use in differential diagnosis of cognitive impairment in the elderly population.

Our data show that EEG outperforms SPECT in several differential diagnoses. We suggest that direct combination of these two modalities is very helpful since they are complementary to each other. Both EEG and SPECT are not the gold standard for the diagnosis of AD and aMCI; however, they are widely used and cost effective. Furthermore, EEG is a non-invasive investigation technique which can be administered many times during the course of the disease. It proved to be more discriminative even at the stage of aSCC. Combining SPECT with EEG should also be subject of further investigations, in order to technically optimize the diagnostic accuracy.

## Author contributions

YH performed the analysis and wrote the first draft of the manuscript, which was revised by AB, RN, FR, ET, and WS. AB, AU, and AJ supervised the work in technical and statistical respects and contributed ideas to how the analysis should be performed and how the results should be presented. NS extracted the EEG data, AL preprocessed the EEG data, JB extracted and pre-processed the SPECT data. MK and JB performed neuropsychological investigations. HZ supervised the work in neuropsychological respects. All of the listed authors have read, commented and approved the manuscript.

### Conflict of interest statement

The authors declare that the research was conducted in the absence of any commercial or financial relationships that could be construed as a potential conflict of interest. The reviewer MD and handling Editor declared their shared affiliation.
