# Supplementary material for: Combining SPECT and Quantitative EEG Analysis for the Automated Differential Diagnosis of Disorders with Amnestic Symptoms
Source: Front Aging Neurosci. 2017 Sep 7;9:290. doi: 10.3389/fnagi.2017.00290 (PMC5594223; doi:10.3389/fnagi.2017.00290)
Supplement: Supplementary file 1 [file DataSheet1.PDF]

Supplementary Material: Combining SPECT and quantitative EEG analysis enhances the accuracy in differential diagnostics of disorders with amnestic symptoms

Yvonne Höller [1,*], Arne C Bathke [2], Andreas Uhl [3], Nicolas Strobl [1], Adelheid Lang [4], Jürgen Bergmann [1], Raffaele Nardone [1,5,6], Fabio Rossini [1], Eugen Trinka [1,5], Harald Zauner [7], Margarita Kirschner [1], Amirhossein Jahanbekam [8], and Wolfgang Staffen [1]

*Correspondence:
Yvonne Höller
y.hoeller@salk.at

1 MEASURES OF INTERACTION

We used the following measures of interaction, also known as connectivity measures:

- **Spectrum:** This contains the auto- and the cross-spectrum, which is the Fourier transform of the cross-covariance function (Murthy, 1963)
- **Direct causality:** Direct causality was developed by Kaminski et al. (2001) to overcome the problem that the directed transfer function does not distinguish between direct and indirect information flows. Direct causality is the only measure that is not computed for each frequency.
- **Transfer function:** This transfer function is related to the non-normalized directed transfer function (Eichler, 2006).
- **Transfer function polynomial:** This is the frequency transform of a polynomial describing the transfer function. The absolute of the squared transfer function polynomial is the non-normalized partial directed coherence (Eichler, 2006).
- **Real valued coherence:** By considering the real part of the complex-valued coherence (Nolte et al., 2004), the result is an ordinary coherence (Schlögl and Brunner, 2008). We will refer to it as coherence.
- **Complex coherence:** By considering the imaginary part of the complex-valued coherence (Nolte et al., 2004), we get complex coherence.
- **Partial coherence:** This is the partial coherence, calculated with an alternative method as provided in the biosig-toolbox. Partial coherence, also known as Gersch causality, was first designed to identify epileptic foci by Gersch and Goddard (1970). The authors proposed that one channel is said to drive the other channels if the first channel explains or accounts for the linear relation between the other two. The real part of the partial coherence was used.
- **Partial directed coherence:** Partial directed coherence as an extended concept of partialized coherence, is a measure of the relative strength of the direct interaction between pairs of regions (Baccalá and Sameshima, 2001).
- **Partial directed coherence factor:** The partial directed coherence factor (Baccalá and Sameshima, 2001) is an intermediate step between partial coherence and partial directed coherence. It adds directionality to partial coherence, but includes instantaneous causality, which is undesirable when examining processes that evolve over time like an epileptic seizure (Schuster and Kalliauer, 2009).
- **Generalized partial directed coherence:** The major advantage of generalized partial directed coherence (Baccalá et al., 2007) over partial directed coherence is its robustness against scaling differences between the signals (Taxidis et al., 2010).
- **Directed transfer function:** Like directed coherence, directed transfer function represents information that flows from one region to another over many possible alternative pathways (Kaminskí and Blinowska, 1991).
- **Direct directed transfer function:** The direct directed transfer function extends the concept of directed transfer function by distinguishing between direct and indirect causal relations of signals (Korzeniewska et al., 2003). As such, the concepts of partial coherence and directed transfer function are combined.
- **full frequency directed transfer function:** The difference between the directed transfer function and the full frequency directed transfer function (Korzeniewska et al., 2003) is that the directed transfer function is normalized by the total frequency content of the considered frequency band, while the full frequency directed transfer function is normalized with respect to all the frequencies in the predefined

frequency interval. As such, the full frequency directed transfer function priorizes those frequencies which contribute the most to the power of the signal (van Mierlo et al., 2011).

- **Geweke's Granger Causality:** This is a modified version of Geweke's Granger Causality (Geweke, 1982), specifically the bivariate version as in Bressler et al. (2007).

By use of the Brain Connectivity Toolbox (Rubinov and Sporns, 2010), we calculated global network parameters from the connection matrices in each frequency range obtained from the multivariate parameters:

- **assortativity:** The assortativity coefficient was calculated for weighted directed connection matrices. This coefficient indicates the correlation between the strengths (weighted degrees) of all nodes on two opposite ends of a link. A positive assortativity coefficient indicates that nodes tend to link to other nodes with similar strength (Newman, 2002; Foster et al., 2010).
- **efficiency:** Efficiency indicates how efficient a network exchanges information. We calculated weighted global efficiency as the average of inverse shortest path length of all directed paths in the network (Latora and Marchiori, 2001; Onnela et al., 2005).
- **clustering coefficient:** The tendency of a network to form tightly connected neighborhoods which would be unlikely to occur in random and uncorrelated networks can be measured by the clustering coefficient (Luce and Perry, 1949; Szabó et al., 2004). The weighted clustering coefficient is the average intensity as given by the geometric mean of all triangles associated with each node (Fagiolo, 2007).
- **modularity:** Modularity indicates the degree to which a network can be subdivided into non overlapping groups of nodes in a way that maximizes the number of within-group edges, and minimizes the number of between-group edges (Newman and Girvan, 2004; Reichardt and Bornholdt, 2006; Leicht and Newman, 2008).
- **transitivity:** With perfect transitivity, a connection between x and y and a connection between y and z implies that x is connected to z, as well. In other words, each pair of reachable nodes in the graph would be connected by an edge. Transitivity is the ratio of triangles to triplets in the network (Wasserman and Faust, 1994; Holland and Leinhardt, 1971; Fagiolo, 2007).

REFERENCES

Murthy V. Estimation of the cross-spectrum. *Ann Math Statist* **34** (1963) 1012–21.

Kaminski M, Ding M, Truccolo W, Bressler S. Evaluating causal relations in neural systems: Granger causality, directed transfer function and statistical assessment of significance. *Biol Cybern* **85** (2001) 145–57.

Eichler M. On the evaluation of information flow in multivariate systems by the directed transfer function. *Biol Cybern* **94** (2006) 469–82.

Nolte G, Bai O, Wheaton L, Mari Z, Vorbach S, Hallett M. Identifying true brain interaction from EEG data using the imaginary part of coherency. *Clin Neurophysiol* **115** (2004) 2292–307.

Schlögl A, Brunner C. BioSig: A free and open source software library for BCI research. *Computer* **41** (2008) 44–50.

Gersch W, Goddard G. Epileptic focus location: spectral analysis method. *Science* **169** (1970) 701–2.

Baccalá L, Sameshima K. Partial directed coherence: a new concept in neural structure determination. *Biol Cybern* **84** (2001) 463–474.

Schuster T, Kalliauer U. Localizing the focus of epileptic seizures using modern measures from multivariate time series analysis. Diploma-Thesis at the Vienna University of Technology (2009).

Baccalá L, Takahashi D, Sameshima K. Generalized partial directed coherence. Sanei S, Chambers J, McWhirter J, Hicks Y, Constantinides A, editors, *Proceedings of the 15th International Conference on Digital Signal Processing (DSP); July 1-4, Wales, UK* (New York: IEEE) (2007), 162–6.

Taxidis J, Coomber B, Mason R, Owen M. Assessing cortico-hippocampal functional connectivity under anesthesia and kainic acid using generalized partial directed coherence. *Biol Cybern* **102** (2010) 327–340.

Kaminskí M, Blinowska K. A new method of the description of the information flow in the brain structures. *Biol Cybern* **65** (1991) 203–210.

Korzeniewska A, Maczak M, Kaminskí M, Blinowska K, Kasicki S. Determination of information flow direction among brain structures by a modified directed transfer function (dDTF) method. *J Neurosci Methods* **125** (2003) 195–207.

van Mierlo P, Carrette E, Hallez H, Vonck K, Van Roost D, Boon P, et al. Accurate epileptogenic focus localization through time-variant functional connectivity analysis of intracranial electroencephalographic signals. *Neuroimage* **56** (2011) 1122–1133.

Geweke J. Measures of conditional linear dependence and feedback between time series. *J Am Stat Assoc* **77** (1982) 304–313.

Bressler S, Richter C, Chen Y, Ding M. Cortical functional network organization from autoregressive modeling of local field potential oscillations. *Stat Med* **26** (2007) 3875–85.

Rubinov M, Sporns O. Complex network measures of brain connectivity: uses and interpretations. *Neuroimage* **52** (2010) 1059–69.

Newman M. Assortative mixing in networks. Phys Rev Lett 89:208701 (2002).

Foster J, Foster D, Grassberger P, Paczuski M. Edge direction and the structure of networks. *Proc Natl Acad Sci USA* **107** (2010) 10815–10820.

Latora V, Marchiori M. Efficient behavior of small-world networks. Phys Rev Lett 87:198701 (2001).

Onnela J, Saramäki J, Kertész J, Káski K. Intensity and coherence of motifs in weighted complex networks. Phys Rev Lett 71:065103(R) (2005).

Luce R, Perry A. A method of matrix analysis of group structure. *Psychometrika* **14** (1949) 95–116.

Szabó G, Alava M, Kertész J. Clustering in complex networks. Ben-Naim E, Frauenfelder H, Toroczkai Z, editors, *Complex Networks* (Berlin, Heidelberg: Springer Berlin Heidelberg) (2004), 139–162.

Fagiolo G. Clustering in complex directed networks. Phys Rev E 76:026107 (2007).

Newman MEJ, Girvan M. Finding and evaluating community structure in networks. Phys Rev E 69:026113 (2004).

Reichardt J, Bornholdt S. Statistical mechanics of community detection. Phys Rev E 2006 76:016110 (2006).

Leicht E, Newman M. Community structure in directed networks. Phys Rev Lett 2008 100:118703 (2008).

Wasserman S, Faust K. *Social Network Analysis: Methods and Applications* (Cambridge: Cambridge University Press) (1994).

Holland P, Leinhardt S. Transitivity in structural models of small groups. *Comparative Group Studies* **2** (1971) 107–124.

2 HEATMAPS

This section contains heatmaps for group contrasts for each single EEG- measure except transfer function and real valued coherence, which are included in the main document. The pixels represent t-values of two-sample t-tests for the respective group comparison, electrode combination and frequency range. A heatmap represents the electrodes in rows and columns.

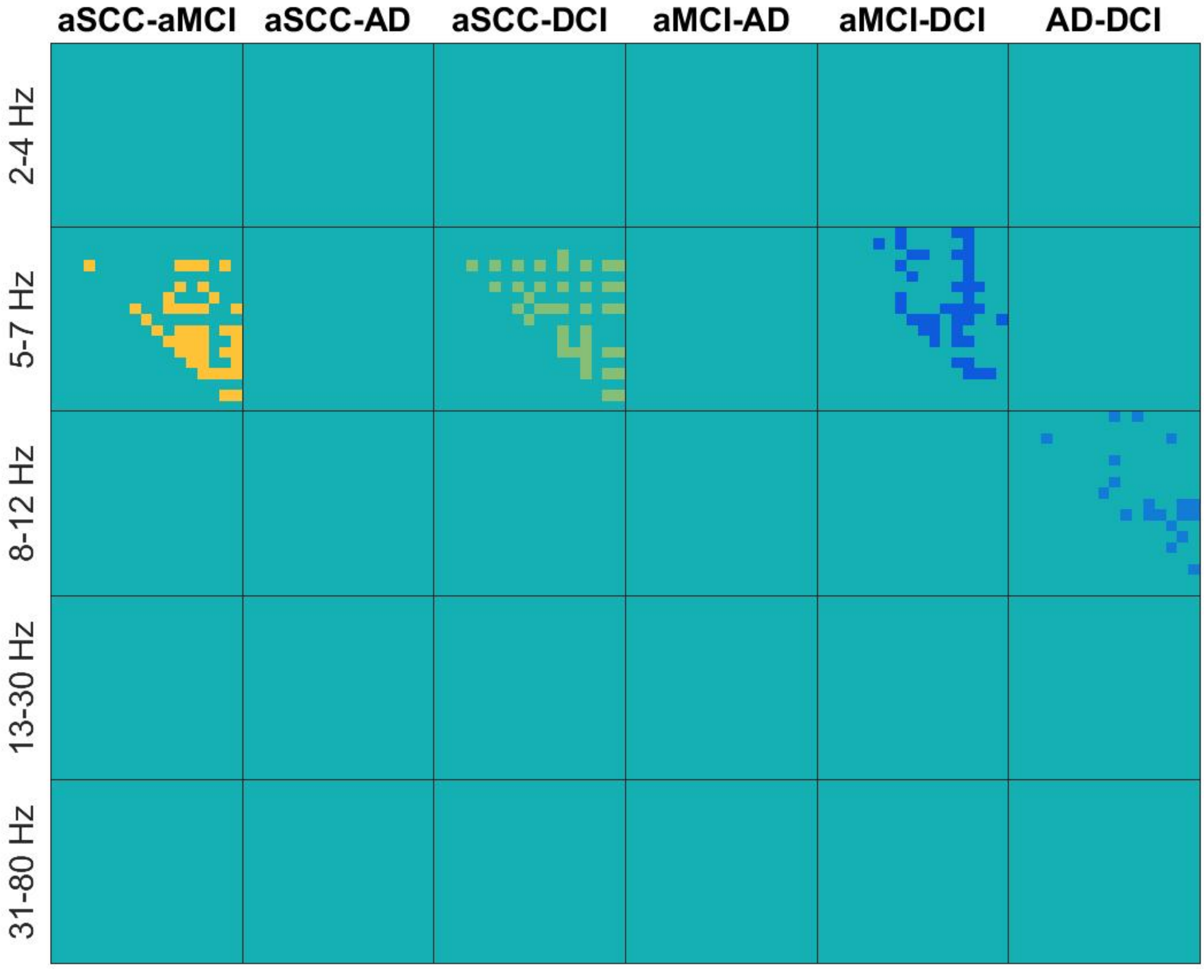

Figure S1. Heatmaps of the t-values of group differences of all electrode × electrode interactions for spectrum, sorted by groups comparisons in columns and frequency ranges in rows. Colours indicate values from -4.11 (dark blue) over zero (green) to +5.24 (yellow). All values that were not included for classification were set to zero. If the first group of the group comparison (e.g. aSCC in aSCC-aMCI) has higher values than the second group, this results in a positive t-value, i.e. yellow colours. Electrodes start from top to bottom and from left to right following the order: F3, F4, C3, C4, P3, P4, O1, O2, F7, F8, T3, T4, T5, T6, Fz, Cz, and Pz. AD: Alzheimer's disease, DCI: depression with cognitive impairment, aMCI: mild cognitive impairment with amnestic symptoms; aSCC: subjective cognitive complaints with amnestic symptoms;

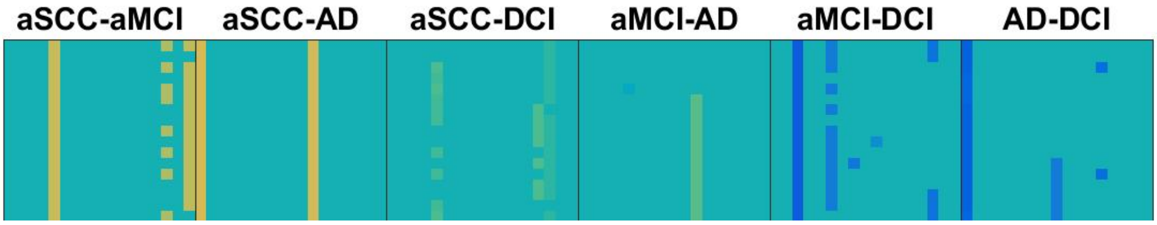

Figure S2. Heatmaps of the t-values of group differences of all electrode × electrode interactions for direct causality, sorted by groups comparisons in columns. Colours indicate values from -4.11 (dark blue) over zero (green) to +5.24 (yellow). All values that were not included for classification were set to zero. If the first group of the group comparison (e.g. aSCC in aSCC-aMCI) has higher values than the second group, this results in a positive t-value, i.e. yellow colours. Electrodes start from top to bottom and from left to right following the order: F3, F4, C3, C4, P3, P4, O1, O2, F7, F8, T3, T4, T5, T6, Fz, Cz, and Pz. AD: Alzheimer's disease, DCI: depression with cognitive impairment, aMCI: mild cognitive impairment with amnestic symptoms; aSCC: subjective cognitive complaints with amnestic symptoms;

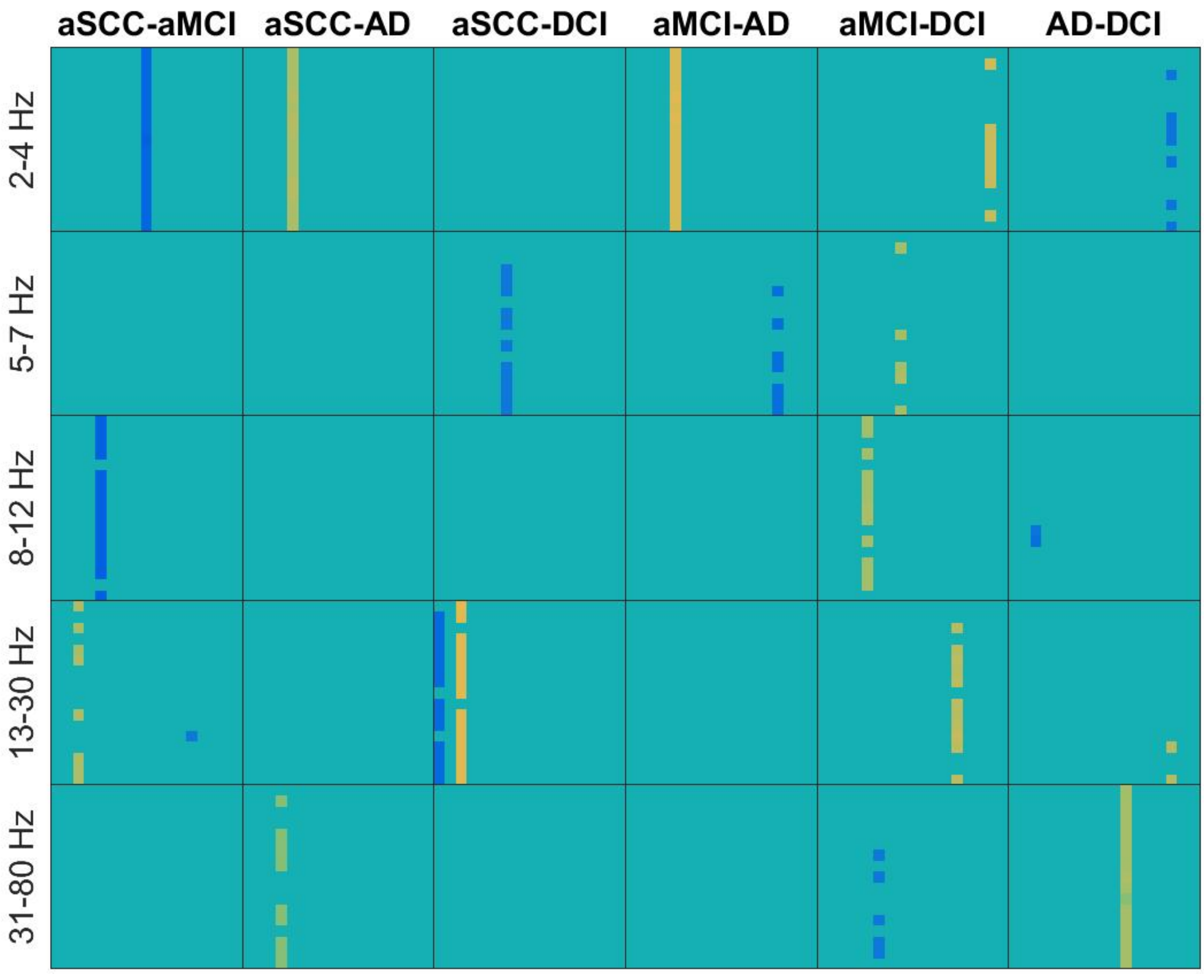

Figure S3. Heatmaps of the t-values of group differences of all electrode × electrode interactions for transfer function polynomial, sorted by groups comparisons in columns and frequency ranges in rows. Colours indicate values from -4.11 (dark blue) over zero (green) to +5.24 (yellow). All values that were not included for classification were set to zero. If the first group of the group comparison (e.g. aSCC in aSCC-aMCI) has higher values than the second group, this results in a positive t-value, i.e. yellow colours. Electrodes start from top to bottom and from left to right following the order: F3, F4, C3, C4, P3, P4, O1, O2, F7, F8, T3, T4, T5, T6, Fz, Cz, and Pz. AD: Alzheimer's disease, DCI: depression with cognitive impairment, aMCI: mild cognitive impairment with amnestic symptoms; aSCC: subjective cognitive complaints with amnestic symptoms;

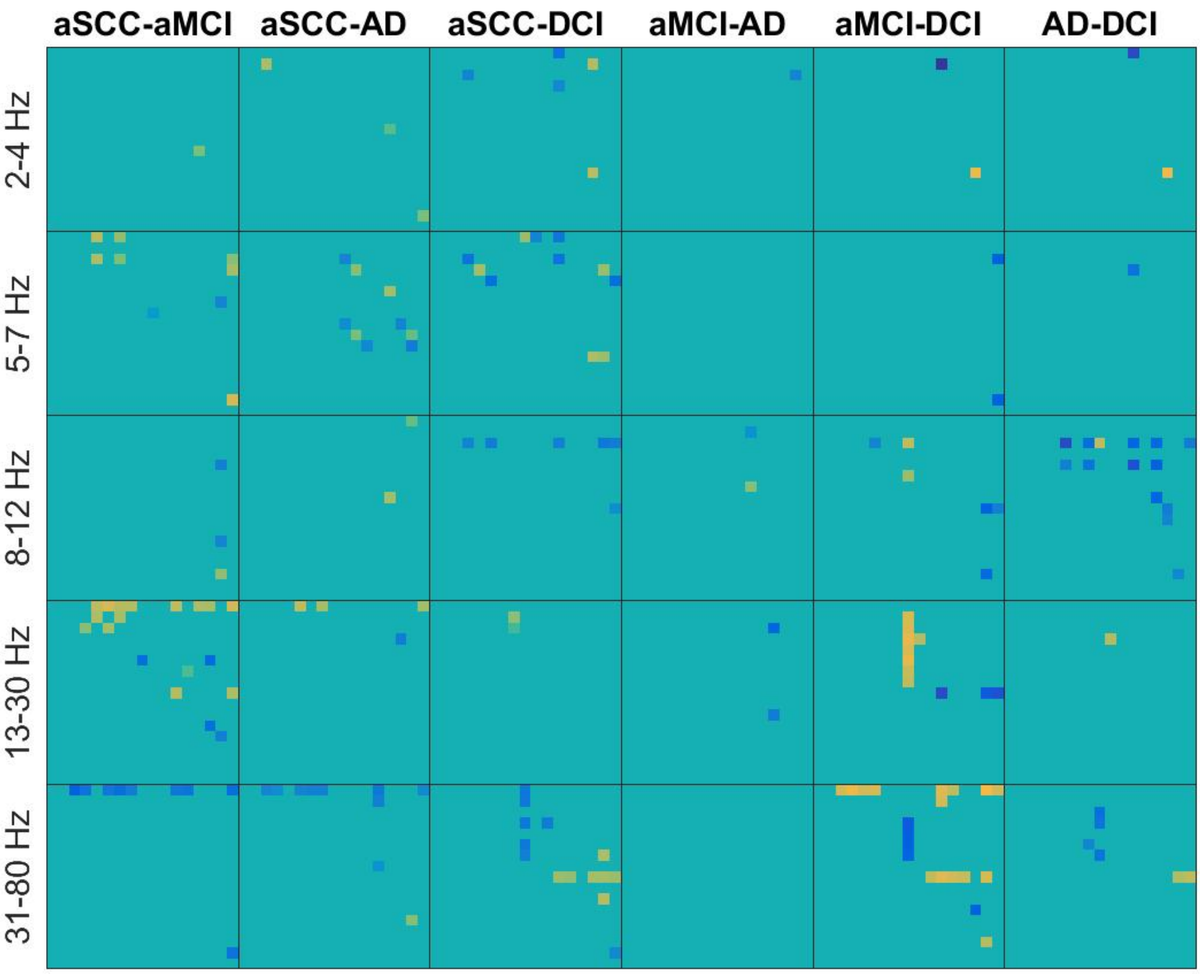

Figure S4. Heatmaps of the t-values of group differences of all electrode × electrode interactions for complex coherence, imaginary part, sorted by groups comparisons in columns and frequency ranges in rows. Colours indicate values from -4.11 (dark blue) over zero (green) to +5.24 (yellow). All values that were not included for classification were set to zero. If the first group of the group comparison (e.g. aSCC in aSCC-aMCI) has higher values than the second group, this results in a positive t-value, i.e. yellow colours. Electrodes start from top to bottom and from left to right following the order: F3, F4, C3, C4, P3, P4, O1, O2, F7, F8, T3, T4, T5, T6, Fz, Cz, and Pz. AD: Alzheimer's disease, DCI: depression with cognitive impairment, aMCI: mild cognitive impairment with amnestic symptoms; aSCC: subjective cognitive complaints with amnestic symptoms;

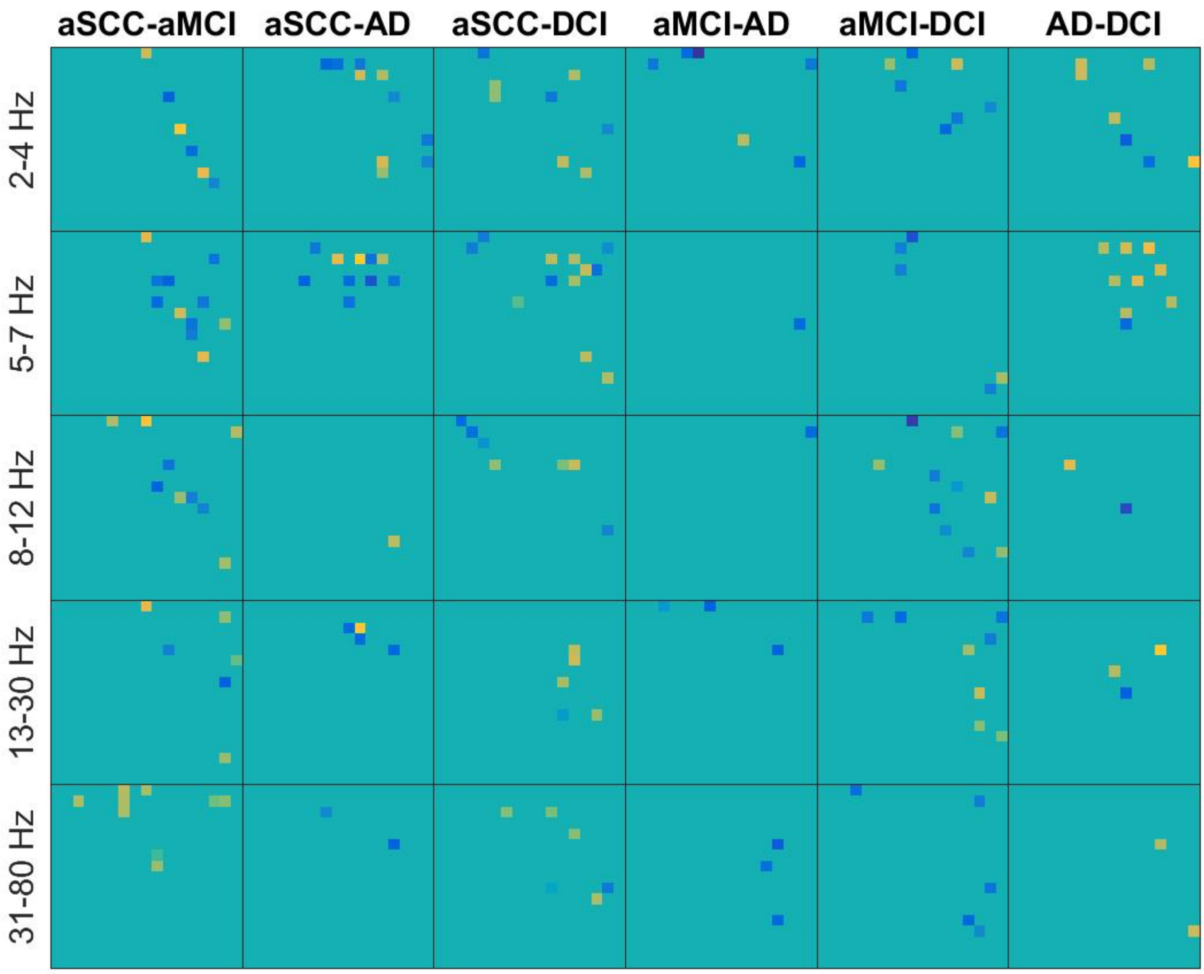

Figure S5. Heatmaps of the t-values of group differences of all electrode $\times$ electrode interactions for partial coherence, sorted by groups comparisons in columns and frequency ranges in rows. Colours indicate values from -4.11 (dark blue) over zero (green) to +5.24 (yellow). All values that were not included for classification were set to zero. If the first group of the group comparison (e.g. aSCC in aSCC-aMCI) has higher values than the second group, this results in a positive t-value, i.e. yellow colours. Electrodes start from top to bottom and from left to right following the order: F3, F4, C3, C4, P3, P4, O1, O2, F7, F8, T3, T4, T5, T6, Fz, Cz, and Pz. AD: Alzheimer's disease, DCI: depression with cognitive impairment, aMCI: mild cognitive impairment with amnestic symptoms; aSCC: subjective cognitive complaints with amnestic symptoms;

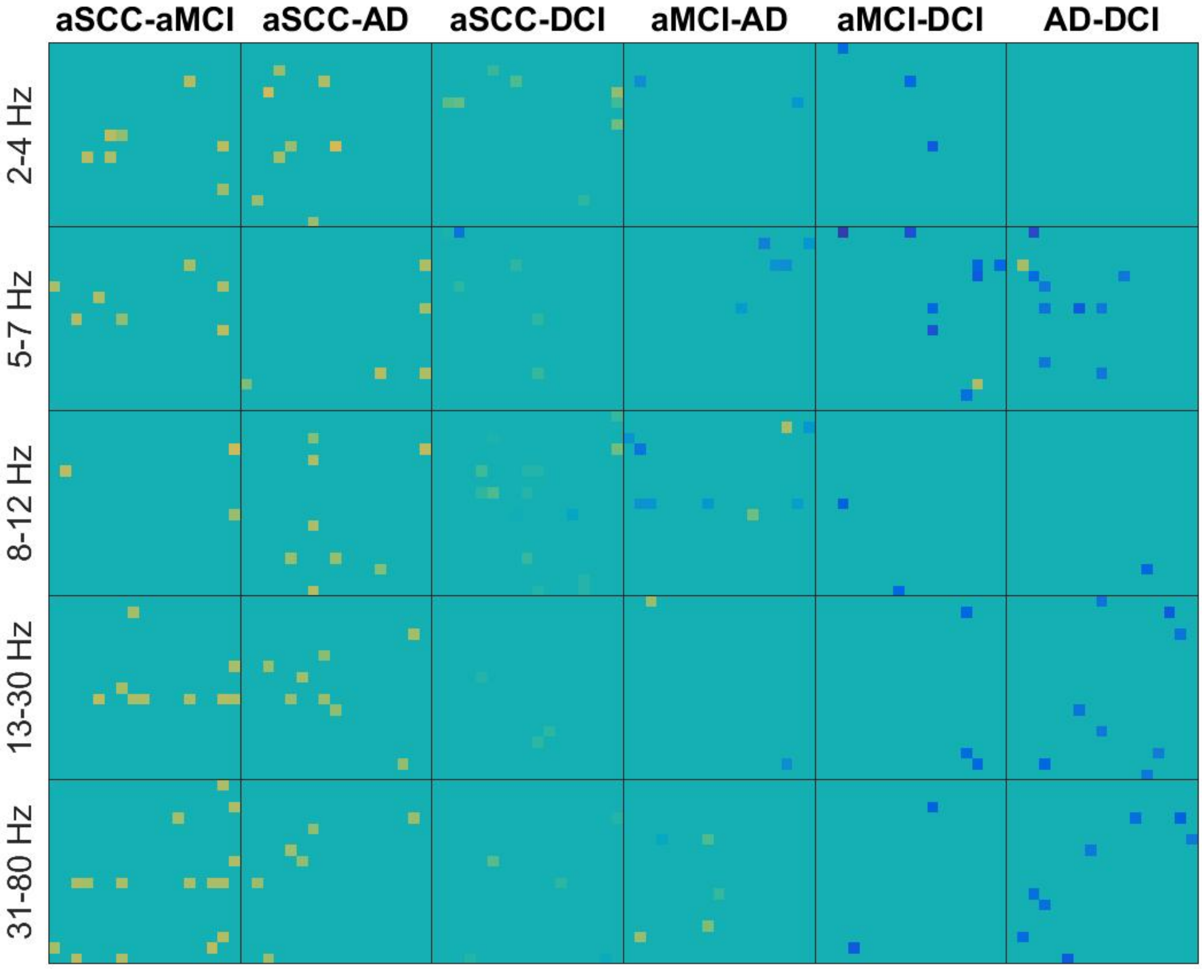

Figure S6. Heatmaps of the t-values of group differences of all electrode × electrode interactions for partial directed coherence, sorted by groups comparisons in columns and frequency ranges in rows. Colours indicate values from -4.11 (dark blue) over zero (green) to +5.24 (yellow). All values that were not included for classification were set to zero. If the first group of the group comparison (e.g. aSCC in aSCC-aMCI) has higher values than the second group, this results in a positive t-value, i.e. yellow colours. Electrodes start from top to bottom and from left to right following the order: F3, F4, C3, C4, P3, P4, O1, O2, F7, F8, T3, T4, T5, T6, Fz, Cz, and Pz. AD: Alzheimer's disease, DCI: depression with cognitive impairment, aMCI: mild cognitive impairment with amnestic symptoms; aSCC: subjective cognitive complaints with amnestic symptoms;

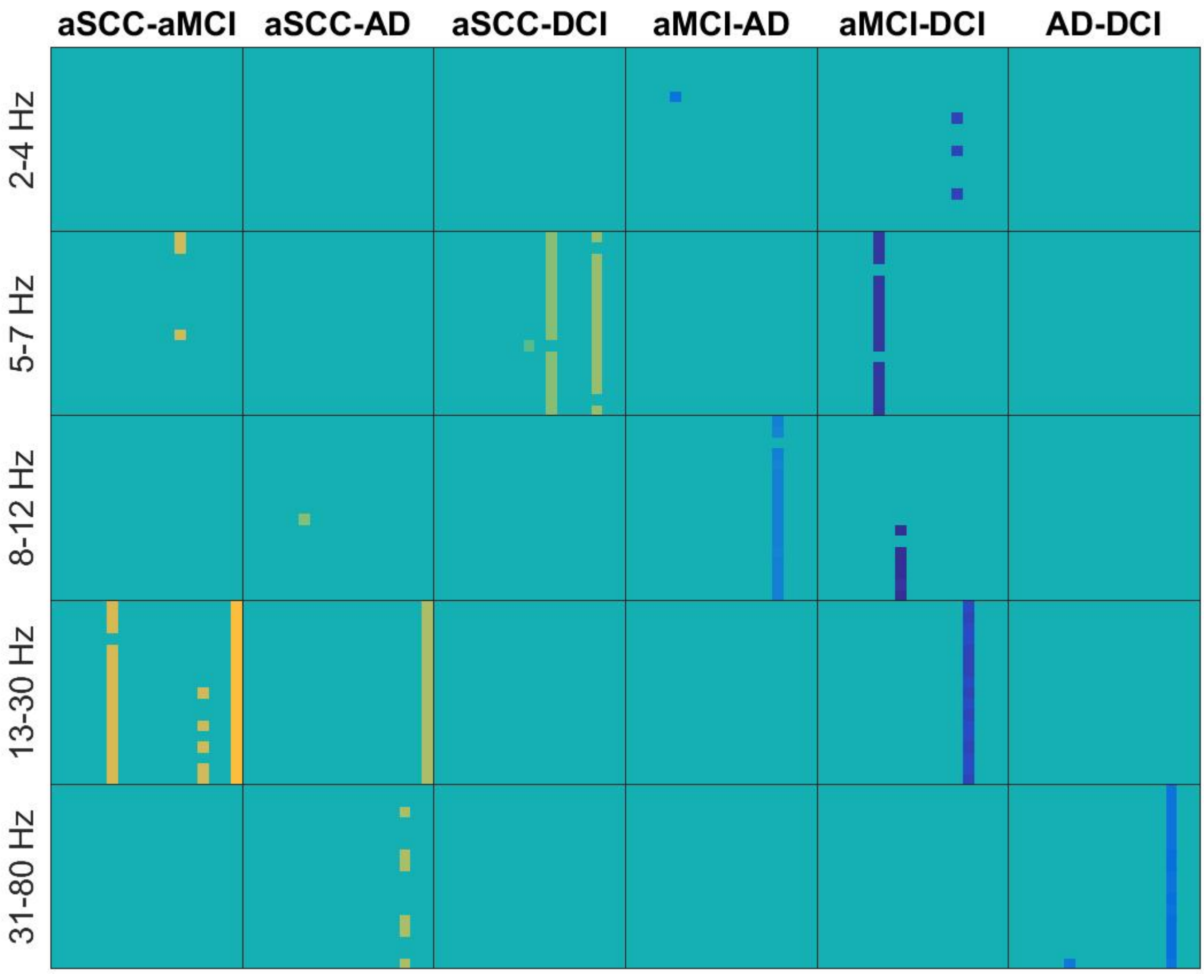

Figure S7. Heatmaps of the t-values of group differences of all electrode $\times$ electrode interactions for partial directed coherence factor, sorted by groups comparisons in columns and frequency ranges in rows. Colours indicate values from -4.11 (dark blue) over zero (green) to +5.24 (yellow). All values that were not included for classification were set to zero. If the first group of the group comparison (e.g. aSCC in aSCC-aMCI) has higher values than the second group, this results in a positive t-value, i.e. yellow colours. Electrodes start from top to bottom and from left to right following the order: F3, F4, C3, C4, P3, P4, O1, O2, F7, F8, T3, T4, T5, T6, Fz, Cz, and Pz. AD: Alzheimer's disease, DCI: depression with cognitive impairment, aMCI: mild cognitive impairment with amnestic symptoms; aSCC: subjective cognitive complaints with amnestic symptoms;

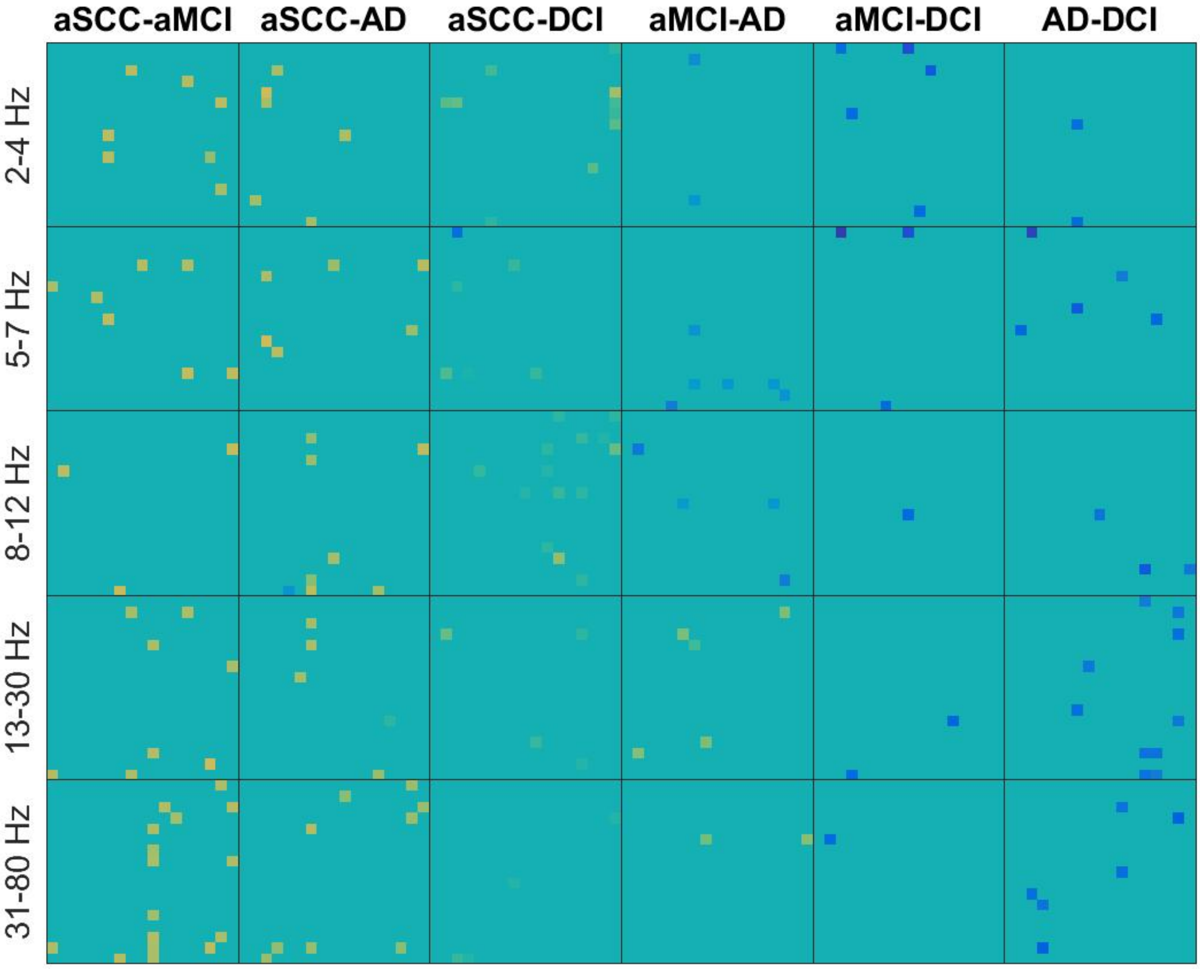

Figure S8. Heatmaps of the t-values of group differences of all electrode $\times$ electrode interactions for generalized partial directed coherence, sorted by groups comparisons in columns and frequency ranges in rows. Colours indicate values from -4.11 (dark blue) over zero (green) to +5.24 (yellow). All values that were not included for classification were set to zero. If the first group of the group comparison (e.g. aSCC in aSCC-aMCI) has higher values than the second group, this results in a positive t-value, i.e. yellow colours. Electrodes start from top to bottom and from left to right following the order: F3, F4, C3, C4, P3, P4, O1, O2, F7, F8, T3, T4, T5, T6, Fz, Cz, and Pz. AD: Alzheimer's disease, DCI: depression with cognitive impairment, aMCI: mild cognitive impairment with amnestic symptoms; aSCC: subjective cognitive complaints with amnestic symptoms;

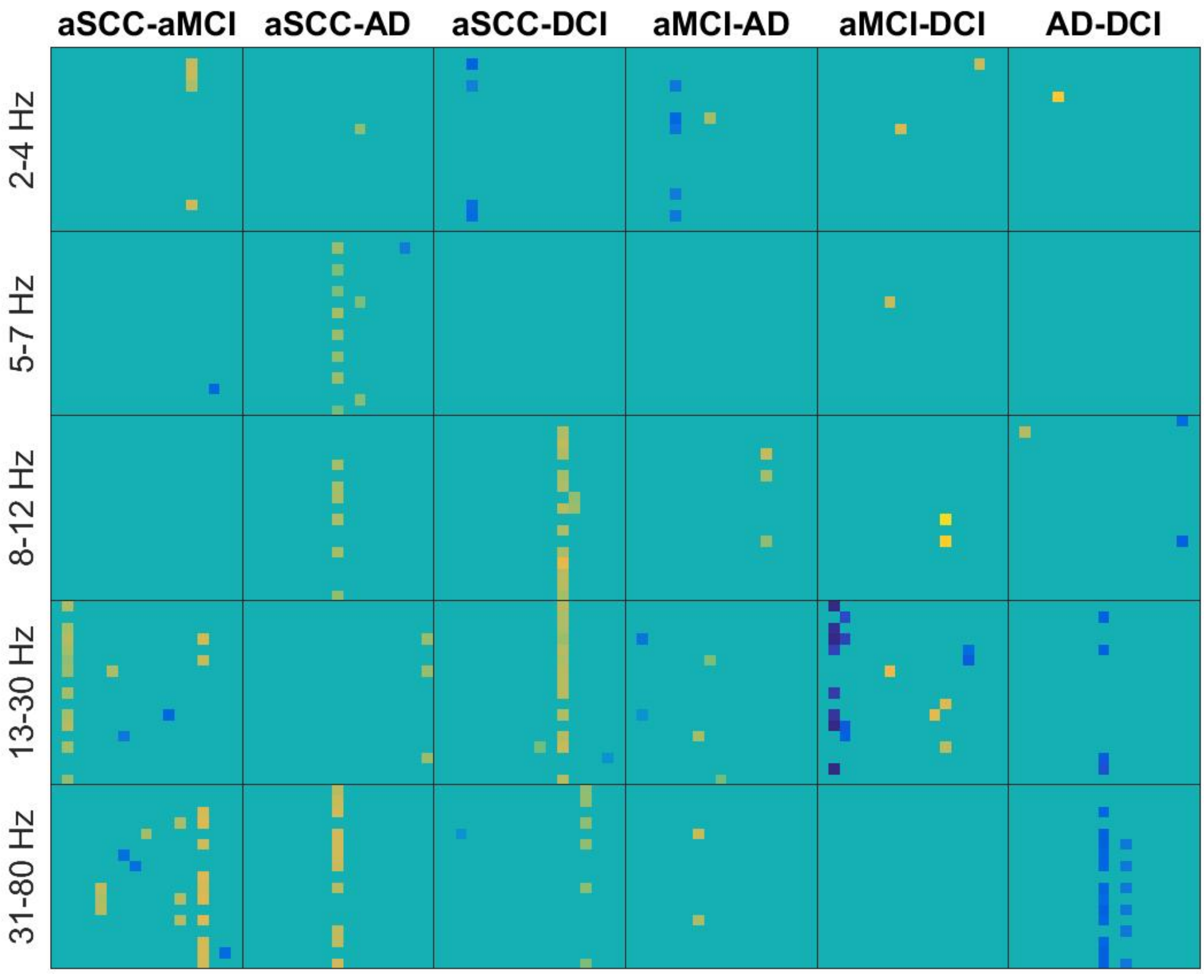

Figure S9. Heatmaps of the t-values of group differences of all electrode × electrode interactions for directed transfer function, sorted by groups comparisons in columns and frequency ranges in rows. Colours indicate values from -4.11 (dark blue) over zero (green) to +5.24 (yellow). All values that were not included for classification were set to zero. If the first group of the group comparison (e.g. aSCC in aSCC-aMCI) has higher values than the second group, this results in a positive t-value, i.e. yellow colours. Electrodes start from top to bottom and from left to right following the order: F3, F4, C3, C4, P3, P4, O1, O2, F7, F8, T3, T4, T5, T6, Fz, Cz, and Pz. AD: Alzheimer's disease, DCI: depression with cognitive impairment, aMCI: mild cognitive impairment with amnestic symptoms; aSCC: subjective cognitive complaints with amnestic symptoms;

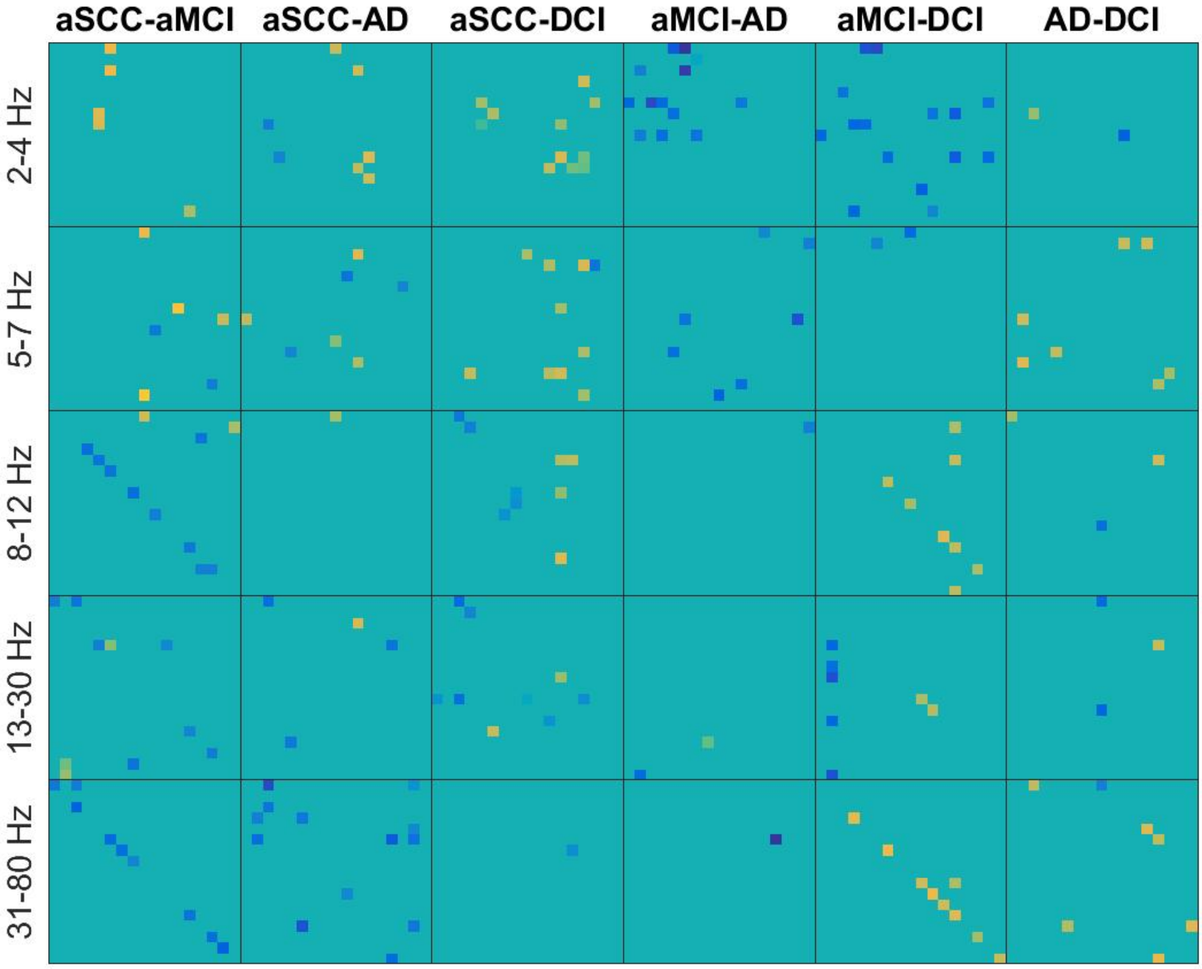

Figure S10. Heatmaps of the t-values of group differences of all electrode × electrode interactions for direct directed transfer function, sorted by groups comparisons in columns and frequency ranges in rows. Colours indicate values from -4.11 (dark blue) over zero (green) to +5.24 (yellow). All values that were not included for classification were set to zero. If the first group of the group comparison (e.g. aSCC in aSCC-aMCI) has higher values than the second group, this results in a positive t-value, i.e. yellow colours. Electrodes start from top to bottom and from left to right following the order: F3, F4, C3, C4, P3, P4, O1, O2, F7, F8, T3, T4, T5, T6, Fz, Cz, and Pz. AD: Alzheimer's disease, DCI: depression with cognitive impairment, aMCI: mild cognitive impairment with amnestic symptoms; aSCC: subjective cognitive complaints with amnestic symptoms;

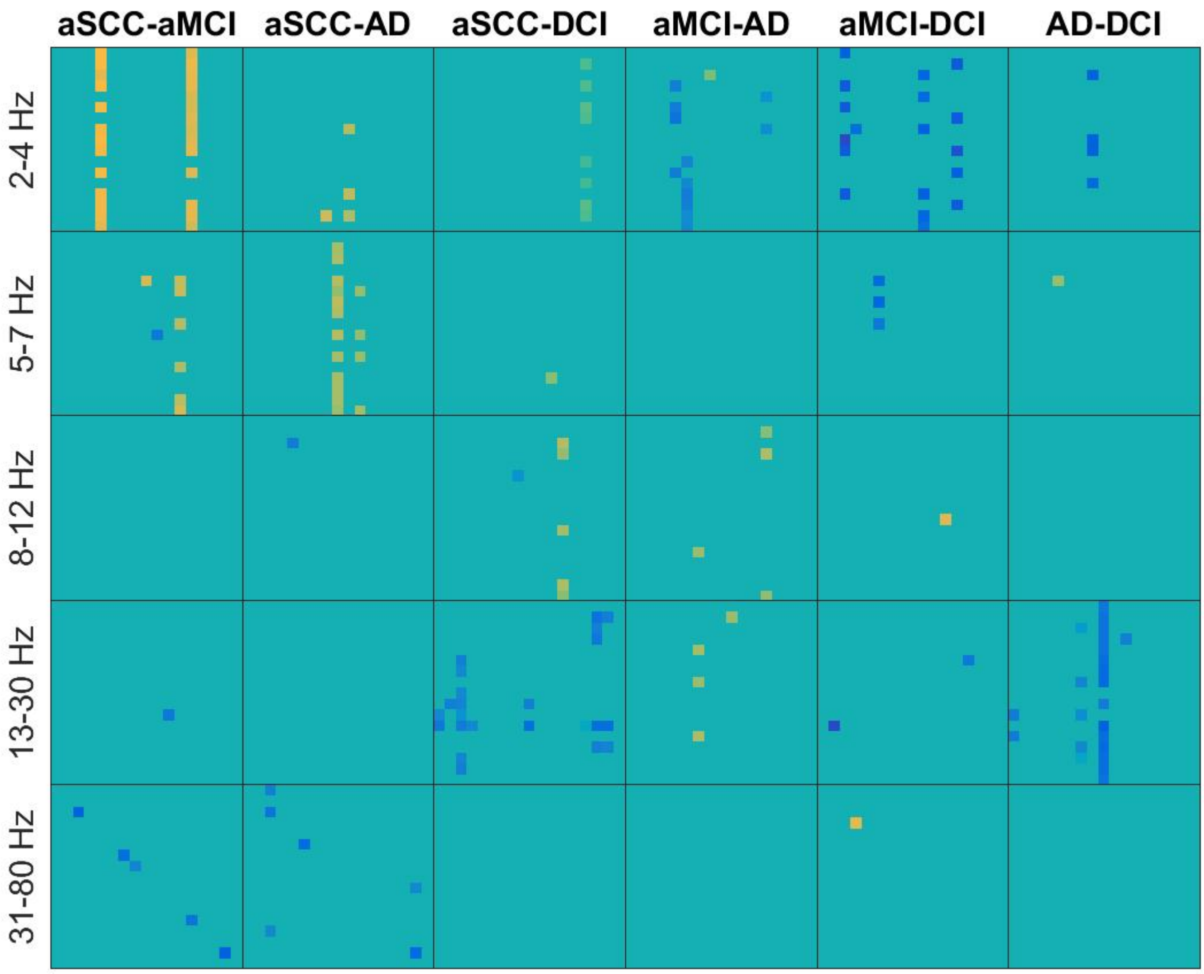

Figure S11. Heatmaps of the t-values of group differences of all electrode $\times$ electrode interactions for full frequency directed transfer function, sorted by groups comparisons in columns and frequency ranges in rows. Colours indicate values from -4.11 (dark blue) over zero (green) to +5.24 (yellow). All values that were not included for classification were set to zero. If the first group of the group comparison (e.g. aSCC in aSCC-aMCI) has higher values than the second group, this results in a positive t-value, i.e. yellow colours. Electrodes start from top to bottom and from left to right following the order: F3, F4, C3, C4, P3, P4, O1, O2, F7, F8, T3, T4, T5, T6, Fz, Cz, and Pz. AD: Alzheimer's disease, DCI: depression with cognitive impairment, aMCI: mild cognitive impairment with amnestic symptoms; aSCC: subjective cognitive complaints with amnestic symptoms;

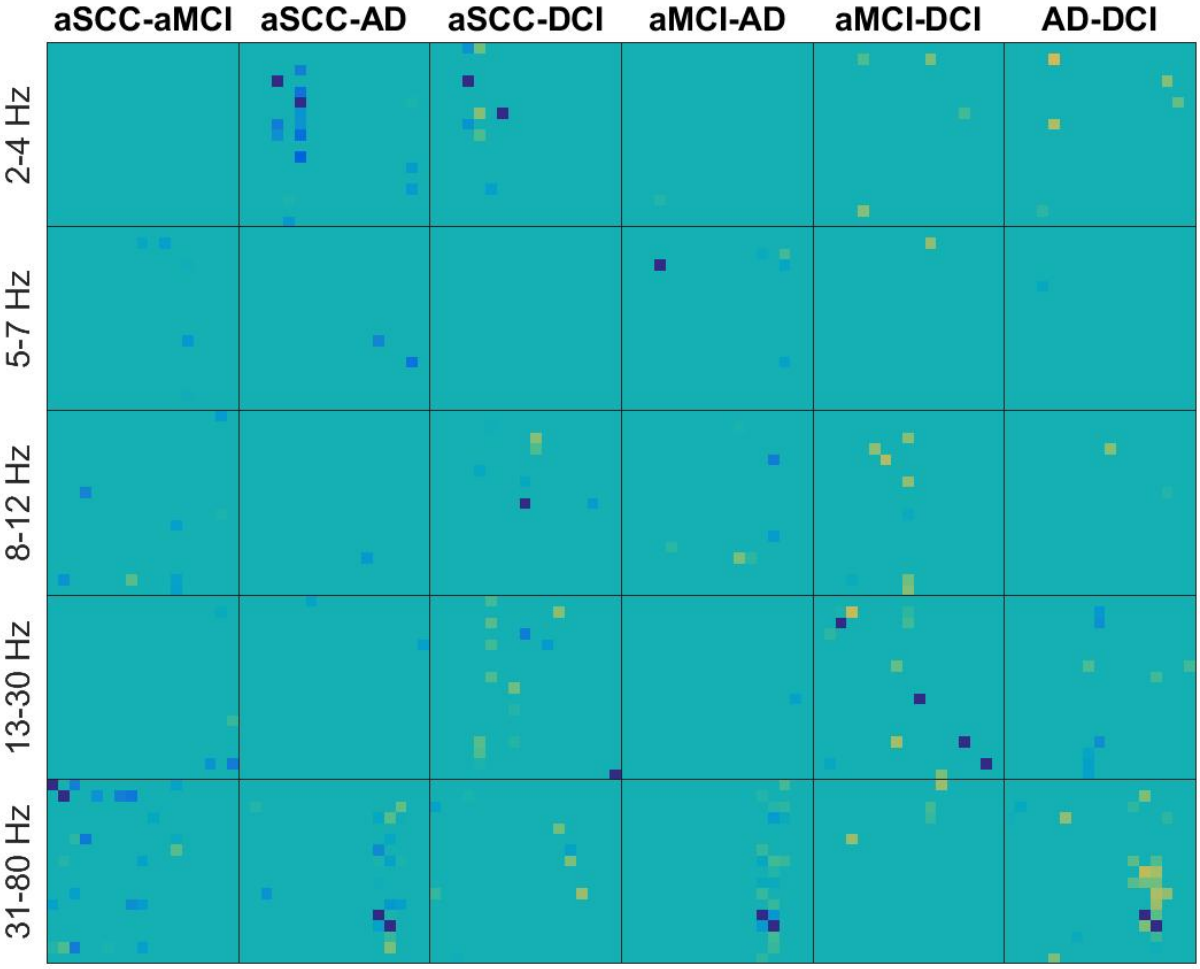

Figure S12. Heatmaps of the t-values of group differences of all electrode $\times$ electrode interactions for Geweke's Granger causality, sorted by groups comparisons in columns and frequency ranges in rows. Colours indicate values from -4.11 (dark blue) over zero (green) to +5.24 (yellow). All values that were not included for classification were set to zero. If the first group of the group comparison (e.g. aSCC in aSCC-aMCI) has higher values than the second group, this results in a positive t-value, i.e. yellow colours. Electrodes start from top to bottom and from left to right following the order: F3, F4, C3, C4, P3, P4, O1, O2, F7, F8, T3, T4, T5, T6, Fz, Cz, and Pz. AD: Alzheimer's disease, DCI: depression with cognitive impairment, aMCI: mild cognitive impairment with amnestic symptoms; aSCC: subjective cognitive complaints with amnestic symptoms;
